# Biochemical and cellular characterization of the CISD3 protein: Molecular bases of cluster release and destabilizing effects of nitric oxide

**DOI:** 10.1016/j.jbc.2024.105745

**Published:** 2024-02-12

**Authors:** Deborah Grifagni, José Malanho Silva, Leonardo Querci, Michel Lepoivre, Cindy Vallières, Ricardo O. Louro, Lucia Banci, Mario Piccioli, Marie-Pierre Golinelli-Cohen, Francesca Cantini

**Affiliations:** 1 Magnetic Resonance Center and Department of Chemistry, University of Florence, Sesto Fiorentino, Italy; 2 CNRS, Institut de Chimie des Substances Naturelles, UPR 2301, Université Paris-Saclay, Gif-sur-Yvette, France; 3 Instituto de Tecnologia Química e Biológica António Xavier (ITQB-NOVA), Universidade Nova de Lisboa, Oeiras, Portugal

**Keywords:** NEET protein, NMR, nitric oxide, iron-sulfur cluster, nitrosative stress

## Abstract

The NEET proteins, an important family of iron-sulfur (Fe-S) proteins, have generated a strong interest due to their involvement in diverse diseases such as cancer, diabetes, and neurodegenerative disorders. Among the human NEET proteins, CISD3 has been the least studied, and its functional role is still largely unknown. We have investigated the biochemical features of CISD3 at the atomic and *in cellulo* levels upon challenge with different stress conditions *i.e.*, iron deficiency, exposure to hydrogen peroxide, and nitric oxide. The redox and cellular stability properties of the protein agree on a predominance of reduced form of CISD3 in the cells. Upon the addition of iron chelators, CISD3 loses its Fe-S clusters and becomes unstructured, and its cellular level drastically decreases. Chemical shift perturbation measurements suggest that, upon cluster oxidation, the protein undergoes a conformational change at the C-terminal CDGSH domain, which determines the instability of the oxidized state. This redox-associated conformational change may be the source of cooperative electron transfer *via* the two [Fe_2_S_2_] clusters in CISD3, which displays a single sharp voltammetric signal at −31 mV *versus* SHE. Oxidized CISD3 is particularly sensitive to the presence of hydrogen peroxide *in vitro*, whereas only the reduced form is able to bind nitric oxide. Paramagnetic NMR provides clear evidence that, upon NO binding, the cluster is disassembled but iron ions are still bound to the protein. Accordingly, *in cellulo* CISD3 is unaffected by oxidative stress induced by hydrogen peroxide but it becomes highly unstable in response to nitric oxide treatment.

NEET family is a class of iron-sulfur proteins occurring in all domains of life (1). In humans, three genes encode for NEET proteins: *CISD1* for mitoNEET, *CISD2* for the nutrient deprivation autophagy factor-1 (NAF-1), also known as CISD2, and *CISD3* for MiNT, also called CISD3. This family of proteins has a conserved domain, called CDGSH, consisting of the consensus sequence [C-X-C-X_2_-(S/T)-X_3_-P-X-C-D-G-(S/A/T)-H], and were initially annotated as zinc-finger proteins (2). Further investigation demonstrated that they actually bind a [Fe_2_S_2_] cluster (3, 4, 5). MitoNEET identified in 2004 (6) as a potential type II diabetes druggable target (7, 8) is bound to the outer mitochondrial membrane *via* a N-terminal transmembrane domain with the main part of the protein, including the Fe-S cluster, which lies in the cytosol. Like mitoNEET, CISD2 is also a transmembrane protein with its cluster protruding into the cytosol but is anchored to the membrane of the endoplasmic reticulum. MitoNEET and CISD2 proteins are structurally similar (9), both are homodimeric proteins binding one cluster per monomer. Differently to both mitoNEET and CISD2, CISD3 is a soluble protein residing in the mitochondrial matrix. CISD3 is a monomer with two CDGSH domains, similar but not equivalent, each binding one [Fe_2_S_2_] cluster (10). Despite their different structure organization, the three human NEET proteins have similar three-dimensional structures (11).

A signature of the NEET proteins is the 3Cys:1His coordination of [Fe_2_S_2_] centers. This unique motif confers cluster lability (12, 13, 14) and facilitates the *in vitro* cluster transfer from NEET proteins to an acceptor protein (15). CISD3 has 127 amino acids and, due to its significant cluster instability, it was necessary to mutate the coordinating histidine residue to a cysteine in order to crystallize it and to solve its X-ray structure (10). When the coordinating His is mutated to Cys, the protein ability to transfer the cluster is abolished, indicating that the Fe-bound His residue is crucial for cluster transfer. The coordinating His is exposed to protonation, suggesting a potential sensing function for this residue (1). *In vitro*, the NEET proteins behave as redox-sensors *i.e.*, they are inert when in the reduced state, whereas in the oxidized state the two clusters can be transferred to apo-acceptor proteins (16, 17, 18).

The interest in these proteins increased significantly when they were found to be potentially implicated in the regulation of reactive oxygen species (ROS), lipid and iron homeostasis, autophagy and apoptosis, and aging (11, 17, 19). However, the exact functions of these proteins are still under discussion and their molecular mechanisms mostly unknown (20). The three human NEET proteins are overexpressed in various types of cancer (11). In support of a possible role of CISD3 in cancer, changes in CpG methylation islands around the CISD3 gene have been observed and were associated with low expression levels in aggressive pediatric brain tumors (21). Recently, CISD3 has been proposed as a regulatory factor in the process of ferroptosis, a nonapoptotic iron-dependent cell death (22). Inhibition of CISD3 cellular expression increases the sensitivity of the cell to inducers of ferroptosis. The use of ferroptosis as a weapon against tumors is of increasing interest, as tumor cells usually exhibit high endogenous oxidative stress levels (23). High intracellular iron concentrations can trigger ferroptosis by enhancing membrane lipid peroxidation, and this can be reverted using iron chelators (24). CISD3 plays a role in mitochondrial function, morphology, and dynamics, particularly in the regulation of the mitochondrial fission/fusion pathway (15, 25). A role of CISD3 in the biogenesis and function of complex I respiratory chain subunit NDUFV2 has also recently been proposed (26). Finally, CISD3 [Fe_2_S_2_] clusters in their reduced [Fe_2_S_2_]^+^ state, are capable of binding nitric oxide (NO) under anaerobic conditions *in vitro* (27, 28). NO is a small radical molecule that has a role in many physiological processes, such as angiogenesis, vasodilation, neurotransmission, inflammatory and immune responses (29, 30, 31). At the cellular level, NO is involved in the control of mitochondrial biogenesis and mitochondrial respiration, cellular signaling, cell proliferation, and death (32). In mammals, nitric oxide synthase enzymes synthesize NO by oxidizing L-arginine to L-citrulline and NO (32). The dysregulation of NO production generates a nitrosative stress, which has been implicated in several diseases (32, 33, 34). At the molecular level, reaction of NO with O_2_, O_2_^−^ and transition metal ions generates a variety of reactive nitrogen species which induce lipid peroxidation, oxidative DNA damages, chemical modification of proteins, and reactions with metal cofactors in metalloproteins (33, 34).

Within this framework, we have delineated for the first time the behavior of CISD3 overexpressed in human HEK-293 cells submitted to oxidative or nitrosative stress or in iron-depletion conditions. We also have analyzed the *in vitro* spectroscopic properties of purified CISD3 binding Fe-S clusters in both reduced and oxidized states and compared the results with mitoNEET and CISD2 proteins. NMR spectroscopy, in combination with UV–visible absorption and electron paramagnetic resonance (EPR) spectroscopies and cyclic voltammetry, provided a full description of the redox states of the clusters of CISD3 as well as of the kinetics of the redox reaction and of the cluster release. The extended NMR assignment of the reduced state of CISD3 (35, 36) opens the possibility to monitor the structural stability of both reduced and oxidized forms and to understand which residues are affected by the oxidation event and which residues are involved in the interaction with nitric oxide.

## Results

### *In cellulo* stability of CISD3: cellular levels are affected by iron depletion

**Figure 1:**
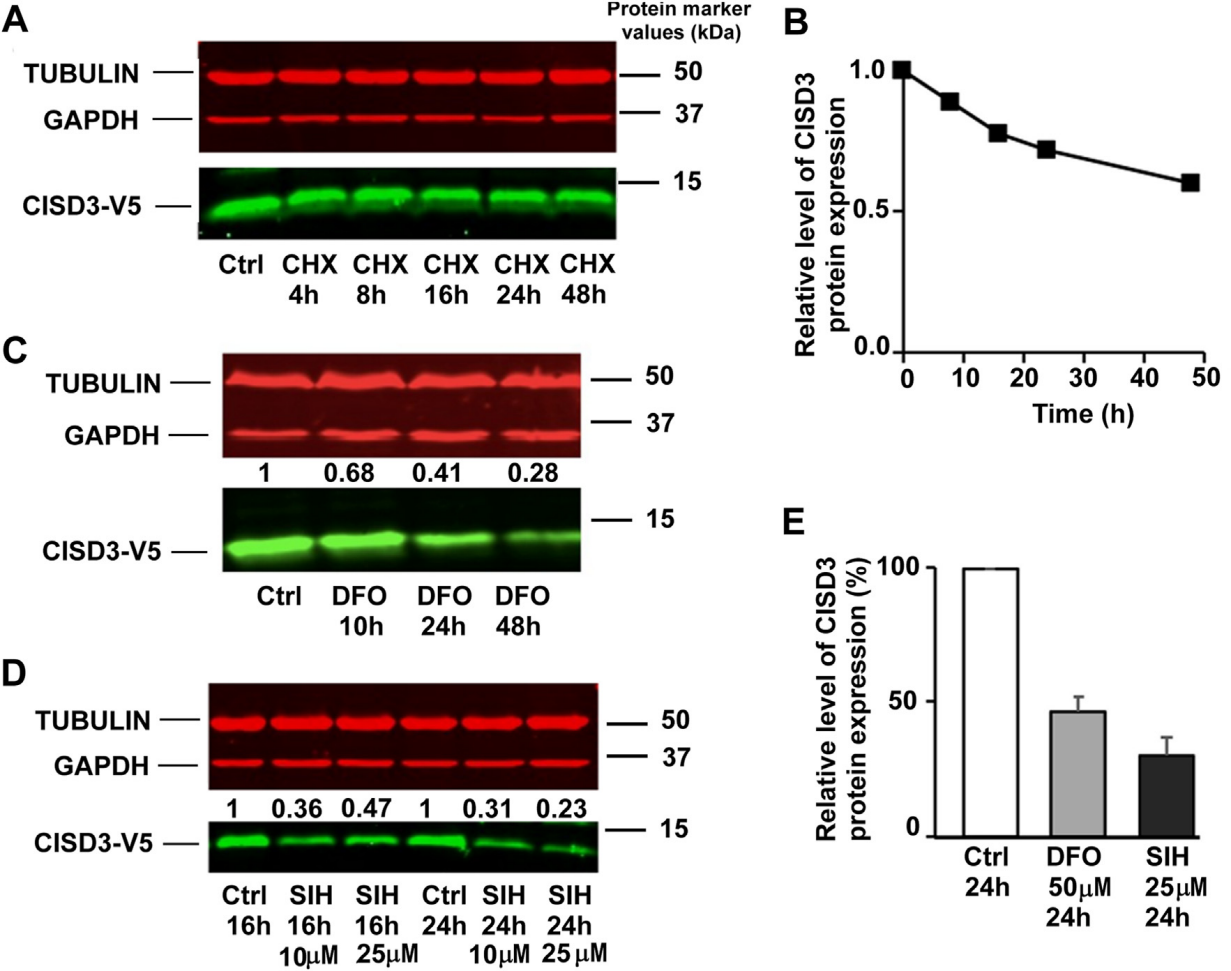
**Effects of CHX and iron chelators treatments on CISD3-V5 protein levels.***A*, CISD3-V5 overexpressing HEK-293 cells were treated with 50 μM CHX for the indicated time periods. CISD3-V5 levels were detected by immunoblotting. *B*, quantification of (*A*) relatively to protein levels in untreated cells (Ctrl) at t = 0. *C*, kinetics data of CISD3-V5 expression in cells treated with DFO at 50 μM for 10, 24, and 48 h. *D*, CISD3-V5 expression in cells treated with SIH at 10 and 25 μM for 16 and 24 h. The reported numbers in Figure 1, *C* and *D* represent the CISD3-V5 protein levels quantification relatively to the untreated control (Ctrl) and corrected from loading variations using the housekeeping proteins ***α***-tubulin and GAPDH. *E*, quantified protein levels after 24 h of exposure to 50 μM DFO and 25 μM SIH treatments. Results are expressed as percentage of untreated control (Ctrl) corrected from loading variations (mean ± SEM, n = 5 (DFO) and n = 3 (SIH). All the experiments were performed at least three times. CHX, cycloheximide; DFO, desferrioxamine; SIH, salicylaldehyde isonicotinoyl hydrazone.

We investigated the half-life of CISD3 in HEK-293 cells using the protein synthesis inhibitor cycloheximide (CHX). First, a V5-tagged CISD3 protein (hereafter CISD3-V5) was overexpressed, as our efforts to detect endogenous or untagged CISD3 with commercially available antibodies were unsuccessful. Cells were then treated with 50 μM CHX to study the decay of CISD3-V5 over a 48-h treatment (Fig. 1, *A* and *B*). The protein level of CISD3-V5 decreased slowly with a half-life of 53.5 ± 2.4 h, indicating that the protein is highly stable. Then, we treated HEK-293 cells overexpressing CISD3-V5 with two different iron chelators, desferrioxamine (DFO), and salicylaldehyde isonicotinoyl hydrazone (SIH), to assess the stability of CISD3-V5 under iron scavengers. Results showed that the protein level of CISD3-V5 decreased by 53.7 ± 5.7% after a 24-h treatment with 50 μM DFO (Fig. 1, *C* and *E*). A slightly larger decrease (69.6 ± 6.2%) was observed with 25 μM SIH (Fig. 1, *D* and *E*). We then considered the possibility that CISD3-V5 cellular level decreases due to mitophagy induction, which may happen under iron limiting conditions (37). However, the presence of 3-methyladenine and chloroquine, respectively to block autophagosome formation and to inhibit lysosomal hydrolytic degradation (38), did not allow any recovery of CISD3-V5 protein level. Similarly, proteasome inhibitors such as MG-132 or bortezomib did not prevent the decrease in CISD3-V5 protein amounts induced by iron chelators (Fig. S1). This indicates that neither mitophagy nor proteasome-dependent proteolysis are responsible for the decrease in CISD3-V5 protein level observed in response to cellular iron depletion.

### *In vitro* stability studies of CISD3

#### CISD3 has only two redox states

**Figure 2:**
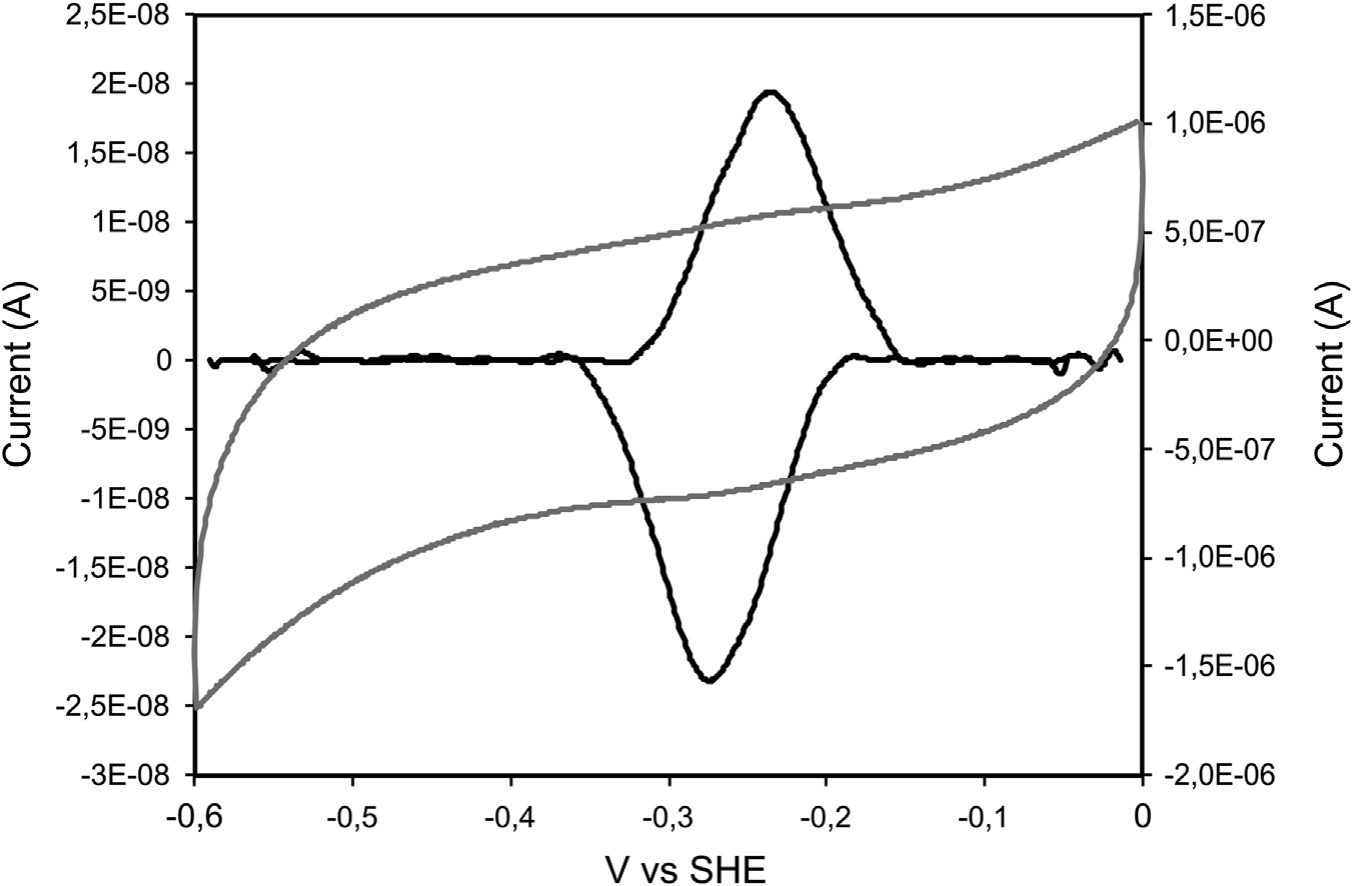
**Protein film voltammetry of CISD3 adsorbed on a PGE electrode at pH7.8and at a scan rate of 50 mV/s.** The raw voltammogram in *gray* is reported *versus* the *right-hand* vertical axis, and the data obtained upon subtraction of the capacitive current is reported in *black versus* the *left-hand* vertical axis. PGE, pyrolitic graphite electrode

The half-life of CISD3-V5 and its sensitivity to cellular iron deprivation led us to characterize CISD3 redox states and to investigate their role in protein stability *via in vitro* studies. We attempted to determine the reduction potentials of the two clusters using protein film voltammetry. We observed a single voltammetric wave, narrower than the 91 mV expected (39), which gives a reduction potential of −33 mV *versus* SHE at pH 7.8 (Fig. 2). Sharp voltammetric signals, as shown in Figure 2, are an indication of positive cooperativity between the redox states of the two clusters (40). The apparent Hill coefficient n of the signal is 1.24 considering the average of anodic and cathodic waves, which is very significant for a protein with two redox clusters (41). A positive cooperativity as observed here requires a redox-associated conformational change in order to overcome the electrostatic repulsion that would normally arise between the two Fe-S clusters as they receive electrons upon reduction. This means that CISD3 is either fully reduced (hereafter 2[Fe_2_S_2_]^+^ CISD3 or reduced CISD3) or fully oxidized (hereafter 2[Fe_2_S_2_]^2+^ CISD3 or oxidized CISD3), and it does not have any mixed redox state in the present conditions. Moreover, the relatively high reduction potential is consistent with the *in cellulo* EPR (Fig. S2), which indicates that at least a significant fraction of the protein in *Escherichia coli* cells, and possibly in human cells, is in the reduced form.

#### Oxidized CISD3 is less stable than reduced CISD3

**Figure 3:**
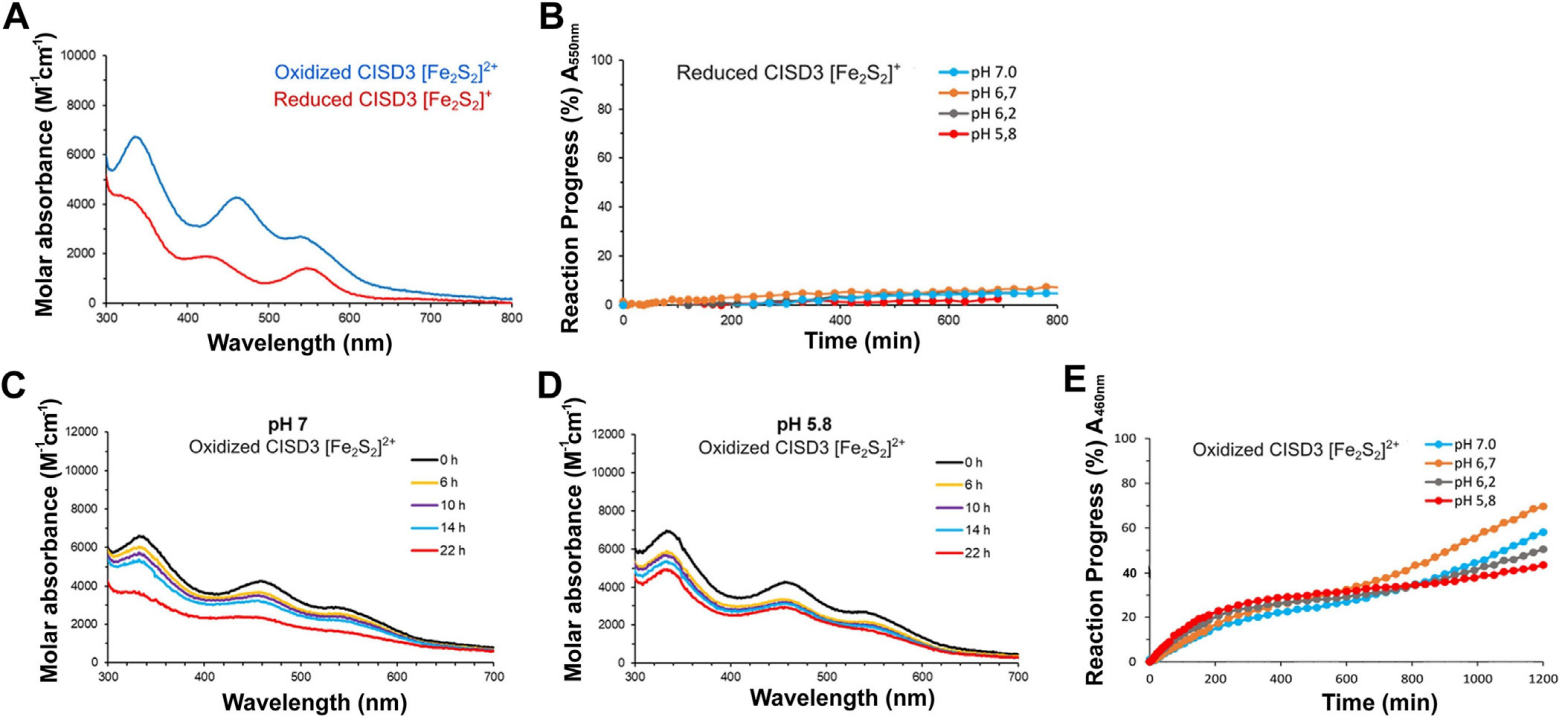
**Stability of the reduced and oxidized CISD3.***A*, UV-visible absorption spectra of the 2[Fe_2_S_2_]^+^ CISD3 (*red line*) and 2[Fe_2_S_2_]^2+^ CISD3 (*blue line*). *B*, reduced CISD3: the intensity of the peak at 550 nm was monitored as a function of the time. Curves describing the pH dependence under anaerobic conditions at 25 °C followed by UV-visible absorption spectroscopy of 2[Fe_2_S_2_]^+^ CISD3 at pH 5.8 (*red*), 6.2 (*gray*), 6.7 (*orange*), and 7 (*blue*). *C* and *D*, oxidized CISD3 under anaerobic conditions: UV-visible absorption spectra at pH 7.0 and pH 5.8, respectively, as a function of the time: 0 h (*black*), 6 h (*yellow*), 10 h (*purple*), 14 h (*blue*), 22 h (*red*). *E*, oxidized CISD3 under anaerobic conditions: the intensity of the peak at 460 nm was monitored as a function of the time. The evolution of the absorbance was expressed as reaction progress.

Oxidized and reduced CISD3 have different UV-visible absorption and 2D ^1^H-^15^N HSQC spectra that can be used to characterize the stability of the holo protein as a function of the oxidation state of the cluster. As shown in Figure 3*A*, the UV-visible absorption spectrum of the oxidized form shows a peak at 460 nm and a shoulder at 550 nm, while the reduced form presents two peaks at 430 nm and at 550 nm, respectively (10). The stability of the two oxidation states was measured by monitoring, at 25 °C, the time evolution of the absorbance bands at 460 nm (for the oxidized form) and at 550 nm (for the reduced form). Under anaerobic conditions, reduced CISD3 is very stable and less than 10% of cluster loss was observed in 24 h, without pH-dependence (Fig. 3*B*). On the contrary, the spectrum of the oxidized protein under anaerobic conditions changes with time (Fig. 3, *C* and *D*). Bands at 460 nm and 550 nm decrease in intensity, indicating a cluster loss. At pH 7.0, a shift of the band at 460 nm to lower wavelengths is also observed, revealing the reduction of the clusters. The latter process is less evident at acidic pH, where the decrease of the intensity of the observed bands is faster (Fig. 3*D*). The reaction progress of the oxidized CISD3 under anaerobic conditions is reported, both cluster loss and cluster reduction are observed as a function of pH and time (Fig. 3*E*).

**Figure 4:**
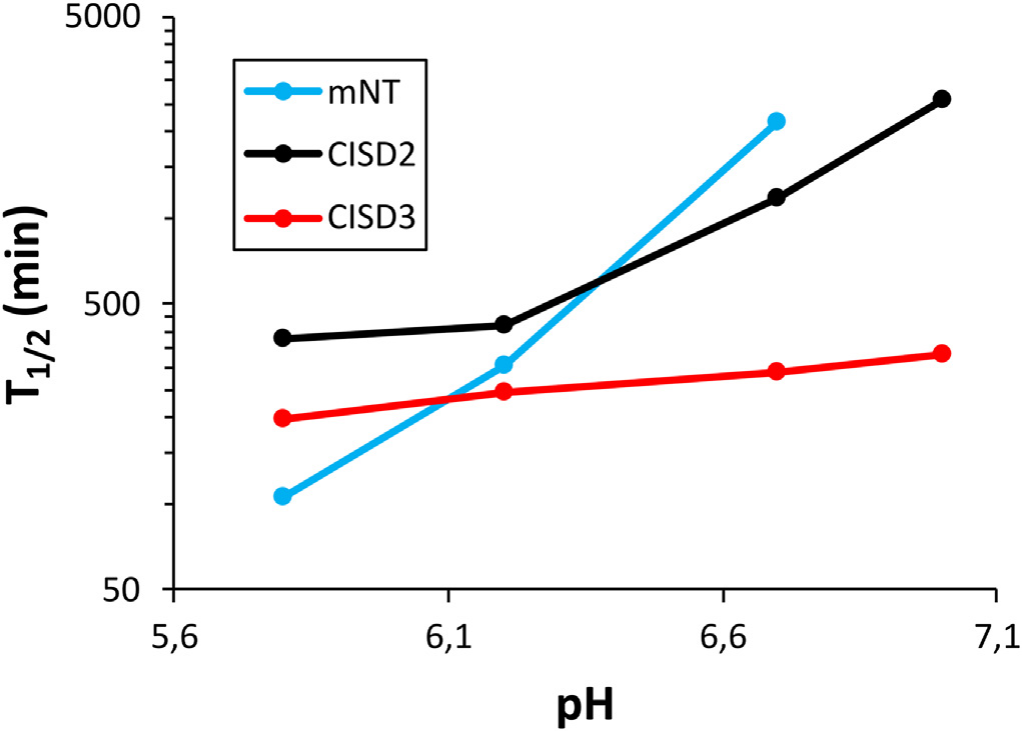
**Comparison between [Fe**_**2**_**S**_**2**_**]**^**2+**^**clusters half-time of CISD1 mitoNEET (mNT, *blue*) CISD2 (*black*), and CISD3 (*red*) at different pH values exposed to air.** The half-time corresponds to the time necessary to lose half of the cluster. Data have been obtained by measuring the 460 nm absorption band using UV-visible absorption spectroscopy.

Cluster loss also happens under aerobic conditions as the instability of the oxidized protein is much higher and was found to slightly increase at acidic pH values (Fig. S3). Moreover, the *in vitro* stability of oxidized holo-CISD3 in the presence of oxygen is significantly lower than the other NEET proteins (Fig. 4). At pH 7.0 the half-life time of oxidized CISD3 under aerobic condition is about five times shorter than CISD2, whereas mitoNEET is at least 10 times more stable than CISD3 (17, 20).

**Figure 5:**
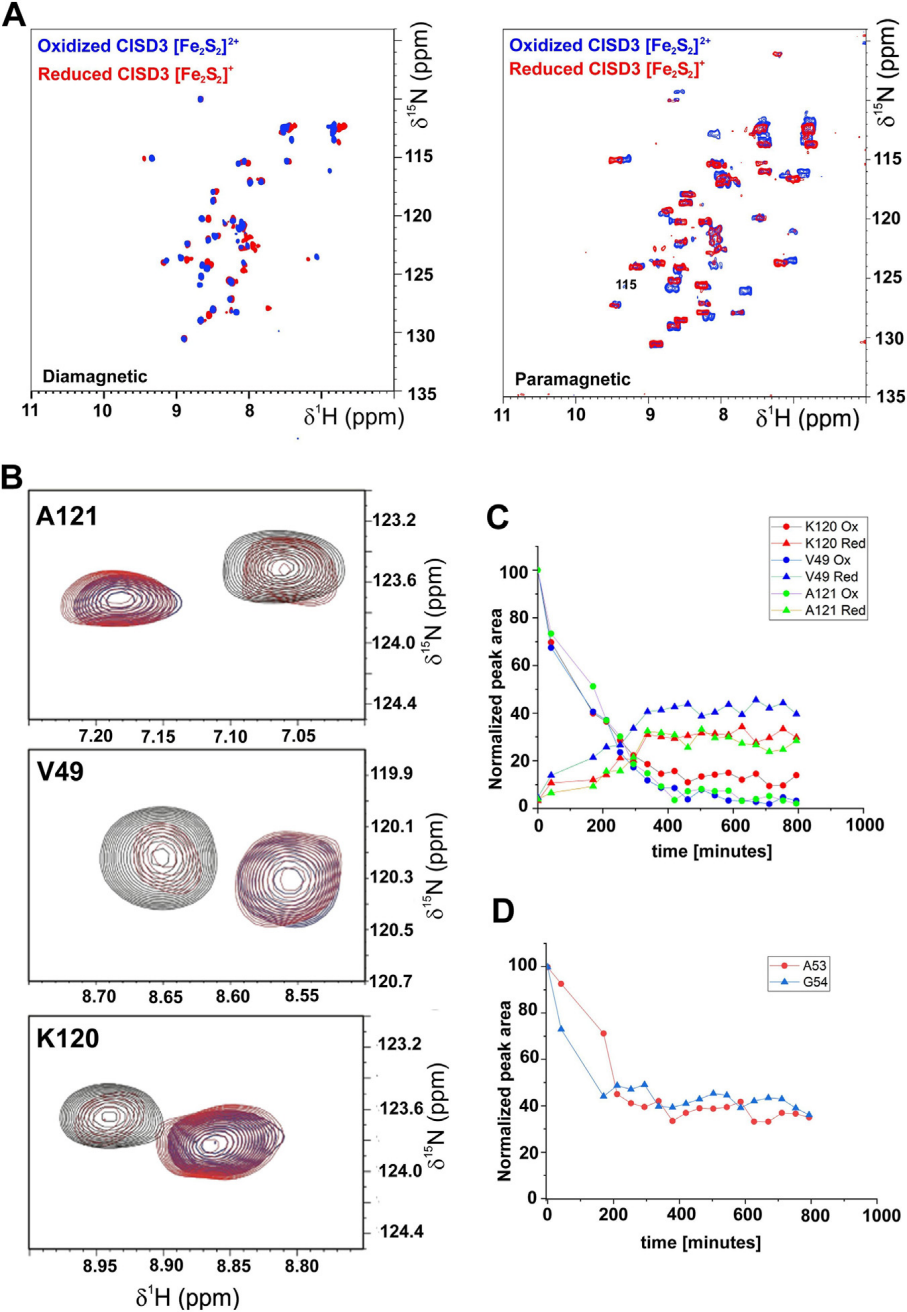
**Stability of the oxidized and reduced CISD3 monitored by NMR.***A*, diamagnetic ^1^H-^15^N-HSQC (*left*) and paramagnetic ^1^H-^15^N-HSQC (*right*) of reduced (*red*) and oxidized (*blue*) CISD3. *B*, insets of the Ala 121, Val 49 and Lys 120 in the ^1^H-^15^N-HSQC spectra of the oxidized CISD3 at different time points: *blue* (0 min), *red* (200 min) and *black* (360 min). *C*, normalized peak areas of residues Lys 120 (*red*), Val 49 (*blue*) and Ala 121 (*green*) in the oxidized and reduced states. *D*, normalized peak areas of residues Ala 53 (*red*) and Gly 54 (*blue*) that only experience a decay in peak area.

The behavior observed for oxidized protein under anaerobic conditions at pH 7.0 suggests the possibility that cluster reduction is coupled to cluster loss, as already observed in mitoNEET (42). This can be better monitored by NMR spectroscopy. The overlay of diamagnetic-type and paramagnetic-type ^1^H-^15^N HSQC spectra of holo-CISD3 for both the oxidized and the reduced states are shown in Figure 5*A*. An extended resonance assignment is available for the reduced form (35) and, due to the small chemical shift differences between the spectra of the two oxidation states it can be extended to the oxidized form by comparison. As already observed by electronic absorption spectroscopy, under anaerobic condition the ^1^H-^15^N-HSQC spectra of the reduced form are stable with time, while for the oxidized form, the resonances of the reduced CISD3 appear at the expenses of corresponding signals of the oxidized form (Fig. 5*B*). In about 300 min, at pH 7, the signals of the oxidized form completely disappear (Fig. 5*C*). About 40% of reduced form is obtained over time. The intensity of peaks whose chemical shift does not change with the oxidation states, such as A53 and G54, provides information on the amount of holo-forms, because the signals of the apo-form are in a different spectral region. Their time evolution shows that 60% of the holo-protein loses clusters and precipitates over time (Fig. 5*D*). Therefore, NMR spectroscopy clearly demonstrates the intrinsic instability of the oxidized CISD3 which loses the clusters even under anaerobic conditions. This event induced the reduction of the remaining holo-protein leading to a mixture of apo- and reduced holo-CISD3. The apo-protein has, in solution, no secondary structure, is unstable, and undergoes protein precipitation. This behavior is also observed when holo-CISD3 is treated with 5 mM ferricyanide and 10 mM of EDTA *i.e.*, the protein partly precipitated and the UV-visible absorption spectrum showed no absorption in the visible part indicating a total loss of the clusters bound to the protein (Fig. S4*A*). In these conditions, the ^1^H-^15^N HSQC spectrum shows that amide resonances collapse in the region typical of an unfolded protein (Fig. S4*B*). The latter behavior demonstrates that the [Fe_2_S_2_] clusters are required for the global folding and stability of CISD3, as previously observed for mitoNEET (19) and CISD2 (20).

#### Atomic level characterization of reduced and oxidized CISD3

**Figure 6:**
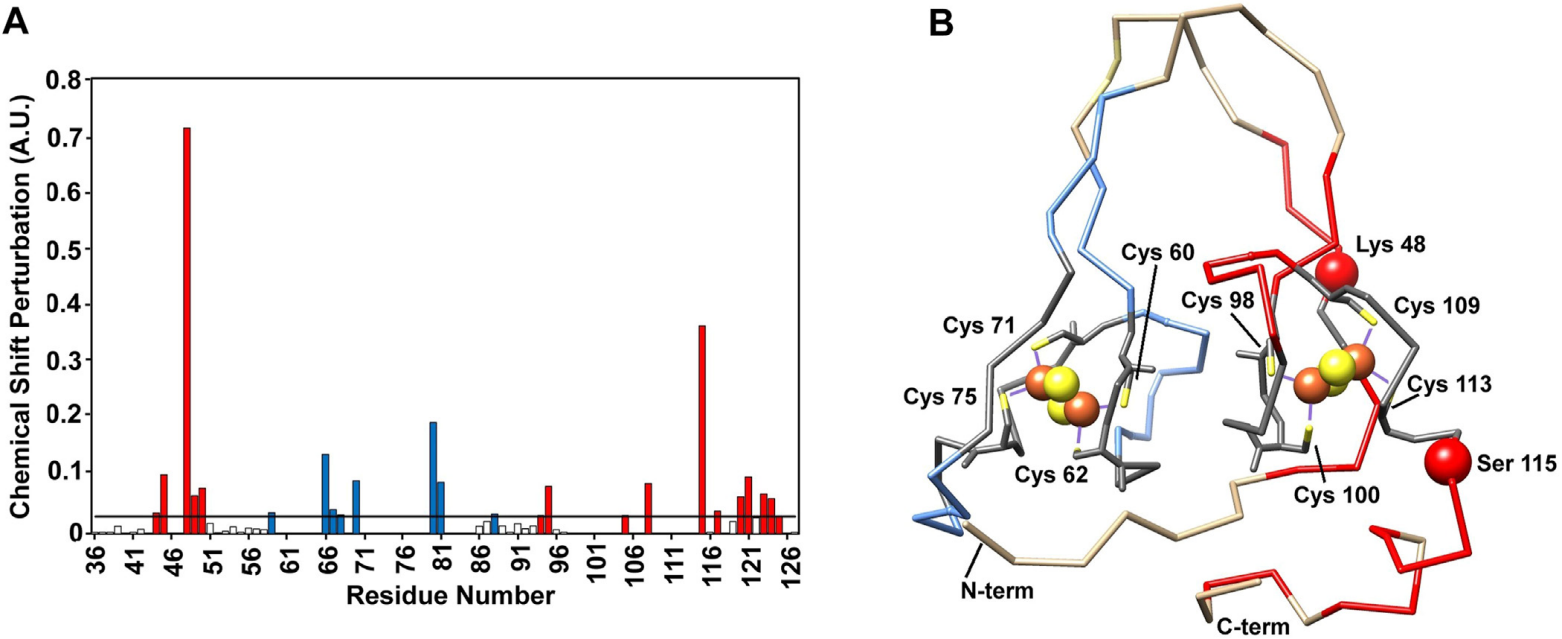
**Chemical shift differences between the reduced and oxidized CISD3.***A*, Garrett plot of redox shift perturbations. On *x*-axis, colored residues are those above the threshold level and belonging to the C-terminal cluster (*red*) or to the N-terminal cluster (*blue*). *B*, CSP highlighted on the Garrett plot are mapped onto the crystal structure of the H75C/H113C mutant of human CISD3 (PDB 6AVJ). The CSP shifts larger than 0.3 ppm are indicated by *spheres*. In *gray* are shown the unassigned residues. The iron and sulfur atoms of the Fe_2_S_2_ cluster are shown as *orange* and *yellow spheres*, respectively, the cysteine residues are labeled with their respective numeration. CSP, chemical shift perturbation; PDB, Protein Data Bank.

**Figure 7:**
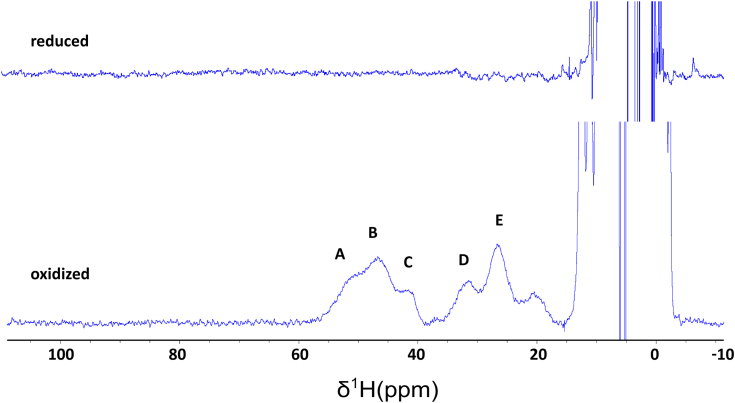
**^1^Honedimensional spectra of reduced (*top*) and oxidized (*bottom*) CISD3.***A*–*E* letters correspond to the protons of cluster coordinating cysteines/histidines.

The NMR assignment of the amide backbone resonances allowed us to perform a per-residue analysis of chemical shift perturbations between the two redox states (Fig. 6*A*). At variance with mitoNEET, where no assignments are available in the 20 amino acids region encompassing the metal coordinating residues and the α helix region (43), in the case of CISD3, tailored NMR approaches and relaxation-based assignments allowed us to identify chemical shift perturbation values also for residues belonging to the regions in proximity of the two clusters (35, 36). Major differences are observed for residues Lys 48 and Ser 115, both next to the C-terminus Fe-S cluster (Ser 115 is two residues after the histidine ligand of the C-terminal cluster, part of the terminal α-helix). Their corresponding residues in the N-terminal part are Lys 86 and Phe 77, respectively. No assignment is available for Phe 77 while Lys 86 experiences a very small perturbation (0.013 ppm) below the threshold level (ca. 0.03 ppm). Differences above 0.1 ppm for residues close to the N-terminal Fe-S cluster are observed only for Lys 66 and Thr 80, whose corresponding residues in the C-terminal, respectively Gln 104 and Val 118, are not assigned. Among the other residues showing chemical shift perturbation above the threshold level, Phe 70 (N-terminal domain) and the corresponding Tyr 108 (C-terminal domain) showed a very similar shift difference (0.092 *versus* 0.088). The paramagnetism of the cluster impedes the identification of NMR signals of residues close to the [Fe_2_S_2_] clusters, even when tailored approaches are used (44, 45), as a consequence we do not have enough data for a reliable comparison among the two cluster sites. However, Figure 6*B* shows that larger perturbations occur in the C-terminal CDGSH domain and, therefore, the two cluster sites might not be equivalent. To provide atomic-level insights into the proximity of the two clusters, we performed ^1^H NMR spectra of the reduced and oxidized CISD3. Paramagnetically shifted ^1^H NMR signals constitute a very unique and sensitive fingerprint for the characterization of the first coordination sphere (44, 45, 46, 47). As shown in Figure 7, oxidized CISD3 shows five hyperfine shifted signals spread between ca. 60 and 25 ppm. The spectrum is very similar to that obtained for oxidized mitoNEET, both in terms of number of signals and of their linewidths (43). The spread of proton signals in mitoNEET and CISD3 is larger (15–60 ppm) than in any of the previously investigated [Fe_2_S_2_]^2+^ proteins, indicating that the unusual 3Cys-1 His coordination provides distinctive and unique features. Signals of the oxidized forms decrease in intensity with time and almost completely disappear in about 16 h, as observed with the other techniques. The large hyperfine shifts indicate that the observed signals belong to the residues that are directly bound to the iron ions, *i.e.*, Cys βCH_2_/αCH and/or His imidazole. The similarity with the spectrum of oxidized mitoNEET suggests that, albeit sequence inequivalence between the two CDGSH domains occurring in CISD3, the geometry and the electronic structure of the ligands of the C-terminal and N-terminal clusters are spectroscopically indistinguishable. Upon protein reduction, no paramagnetic spectrum can be observed. Again, the same behavior of ^1^H paramagnetic NMR spectrum has been observed for mitoNEET (43), thus confirming that the similarity between mitoNEET and CISD3 in terms of coordination sphere and electronic properties holds for both oxidation states.

### Effects of H_2_O_2_ and NO on CISD3 protein stability

#### Decreased expression of the CISD3-V5 protein in cells exposed to a chemical NO donor

**Figure 8:**
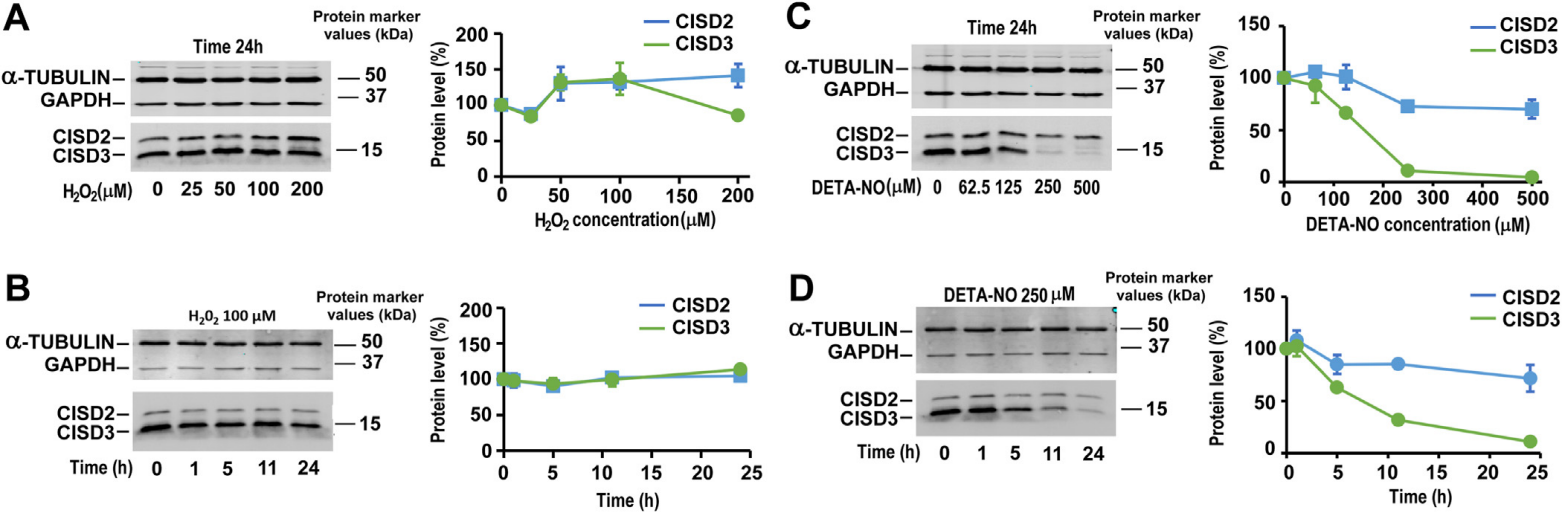
**Evolution of CISD3 and CISD2 protein levels under oxidative and nitrosative cellular stress in V5-CISD3 HEK cells.** On the *left side* is shown a representative Western blot using α-tubulin and GADPH as loading controls. The graph on the *right side* shows protein quantification from at least three similar independent experiments (percentage of untreated control, mean ± SEM). *A*, cells were cultured for 24 h in the presence of increasing concentration of H_2_O_2_ (25, 50, 100, and 200 μM). *B*, time-dependent analysis of CISD3 and CISD2 protein level in the presence of 100 μM H_2_O_2_. *C*, dose-effect of increasing concentration of DETA-NO (62.5, 125, 250, and 500 μM) for 24 h. *D*, kinetics variations in CISD3 and CISD2 protein levels at 250 μM DETA-NO. NO, nitric oxide.

A recent study has proposed that CISD3 is involved in the control of mitochondrial ROS levels and cell resistance to ROS (15). Moreover, CISD3 clusters can bind NO *in vitro* and NO-bound clusters become resistant to redox transition (28, 48), but how NO affects CISD3 protein level *in cellulo* is unknown. Therefore, we investigated the effects of H_2_O_2_ and NO on the CISD3-V5 protein overexpressed in HEK-293 cells. Cells were treated with increasing concentrations of H_2_O_2_ and DETA-NO, a long-lived NO donor mimicking inducible NO synthase 2 activity (t_1/2_ = 20 h at 37 °C and pH 7.4), for 24 h (see Material and Methods for details). At the highest concentrations tested (250 and 500 μM), steady-state concentrations of NO released by DETA-NO are in the micromolar range. Kinetics experiments were also performed using 100 μM H_2_O_2_ and 250 μM DETA-NO. CISD3-V5 protein levels were quantified by immunoblotting, together with CISD2 for comparison. As reported in Figure 8, *A* and *B*, H_2_O_2_ treatments did not affect CISD3 and CISD2 protein levels. By contrast, NO induced a dramatic and dose-dependent reduction in CISD3 levels, leading to an almost complete loss of the protein after a 24-h treatment with 250 μM DETA-NO (Fig. 8, *C* and *D*). CISD2 was much more resistant than CISD3 to NO and underwent only a 25% decrease in its level after 24 h in the presence of 250 or 500 μM DETA-NO. There is thus a specificity in CISD3 instability in the presence of NO compared to CISD2 under the same conditions in cells.

#### *In vitro* study of the effect of H_2_O_2_

**Figure 9:**
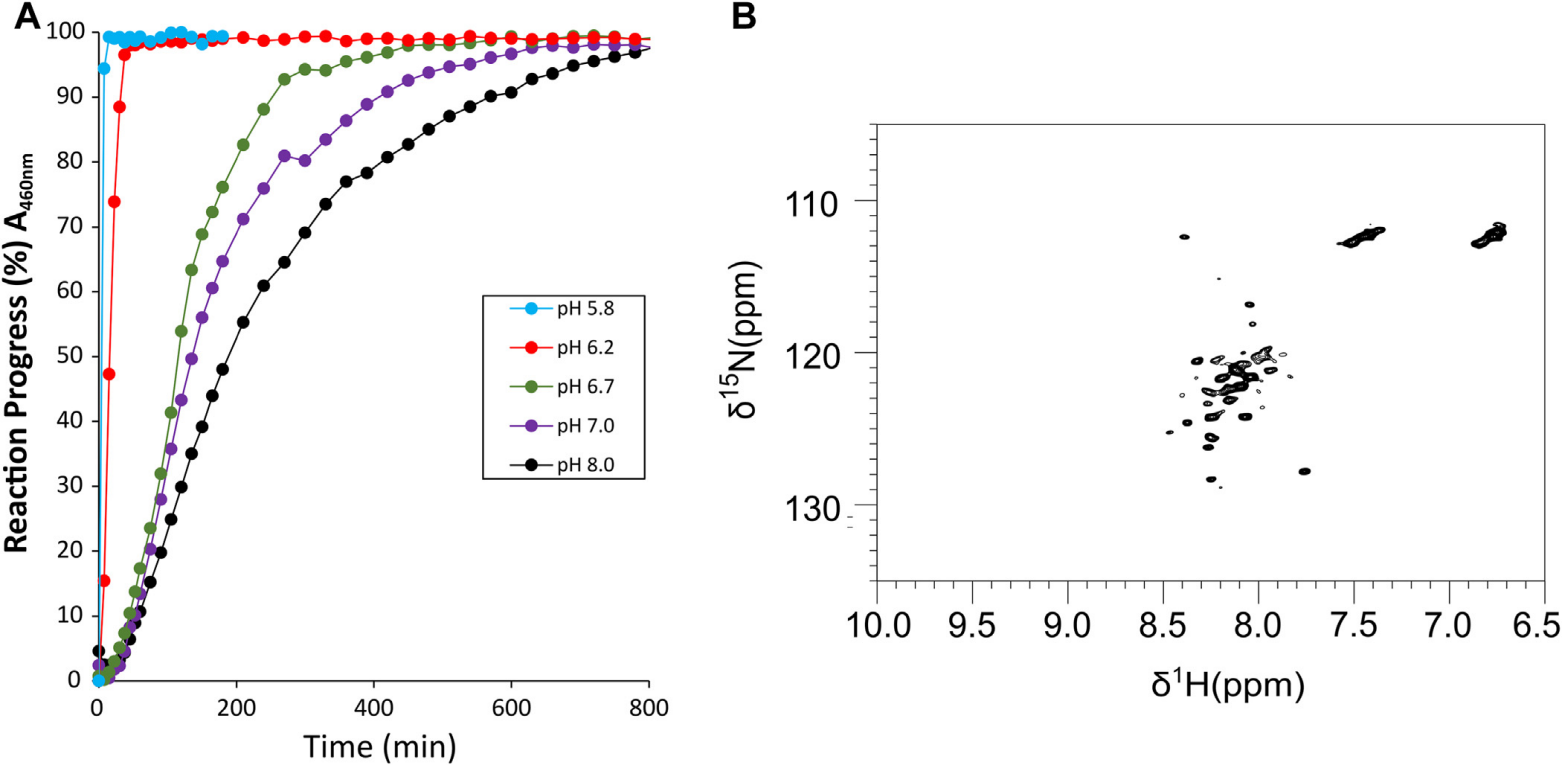
**CISD3 sensitivity to H**_**2**_**O**_**2**_***in vitro*.***A*, cluster loss reactions with 20 μM protein and 250 μM H_2_O_2_ were performed at 25 °C under anaerobic conditions and followed by UV-visible absorption spectroscopy. The intensity of the peak at 460 nm was monitored as a function of the time at various pH values. The reaction buffer (50 mM Tris–HCl or Bis–Tris with 100 mM NaCl) was at pH 5.8 (*blue*) 6.2 (*red*), 6.7 (*green*), 7.0 (*purple*), and 8.0 (*black*), respectively. *B*, ^1^H-^15^N-HSQC spectrum of CISD3 240 min after the addition of four equivalents of hydrogen peroxide (H_2_O_2_). Protein concentration was 600 μM in 20 mM Tris–HCl, 150 mM NaCl buffer at pH 7.5.

We monitored the stability of CISD3 clusters in the presence of a large excess of H_2_O_2_ (250 μM) at different pH values, under anaerobic conditions, by using UV-visible absorption spectroscopy (Fig. 9*A*). Addition of H_2_O_2_ on reduced NEET proteins *in vitro* is known to oxidize the cluster and we might expect that the same occurs *in cellulo* (49). Then, the *in vitro* studies were performed only on the oxidized protein. At pH lower than 6.7, CISD3 clusters were highly labile upon H_2_O_2_ treatment, with a half-life shorter than 15 min. At higher pH values, the destabilization of the clusters by H_2_O_2_ treatment was less efficient, with a t_1/2_ in the range of 100 to 200 min for pH values between 6.7 and 8.0 (Table 1). NMR has also followed the induced oxidative stress. When two equivalents of H_2_O_2_ are added to the oxidized CISD3 under anaerobic conditions, a significant decrease of peak intensity is observed for the signals of the oxidized form, together with the appearance of new peaks in the 6.5 to 8.5 ppm region, typical of unfolded polypeptide chains. When four equivalents of H_2_O_2_ are added, only the spectrum of the unfolded protein appeared (Fig. 9*B*). The oxidative stress induced by H_2_O_2_ results in a complete loss of the two clusters and of the secondary structure of CISD3 *in vitro*.

**Table 1:** Comparison of the half-time of [Fe_2_S_2_]^2+^ clusters of mitoNEET, CISD2 and CISD3 (20 μM protein) exposed to 250 μM H_2_O_2_ at 25 °C under anaerobic conditions at different pH

PH	6.2	6.7	8.0
mitoNEET	T_1\2_≈15 – 50 min	T_1\2_≈50 min	Not sensitive
CISD2	T_1\2_≈12 – 15 min	T_1\2_≈50 – 60 min	Not sensitive
CISD3	T_1\2_≈15 – 20 min	T_1\2_≈110 – 120 min	T_1\2_≈190 – 200 min

#### *In vitro* study of the effect of NO

**Figure 10:**
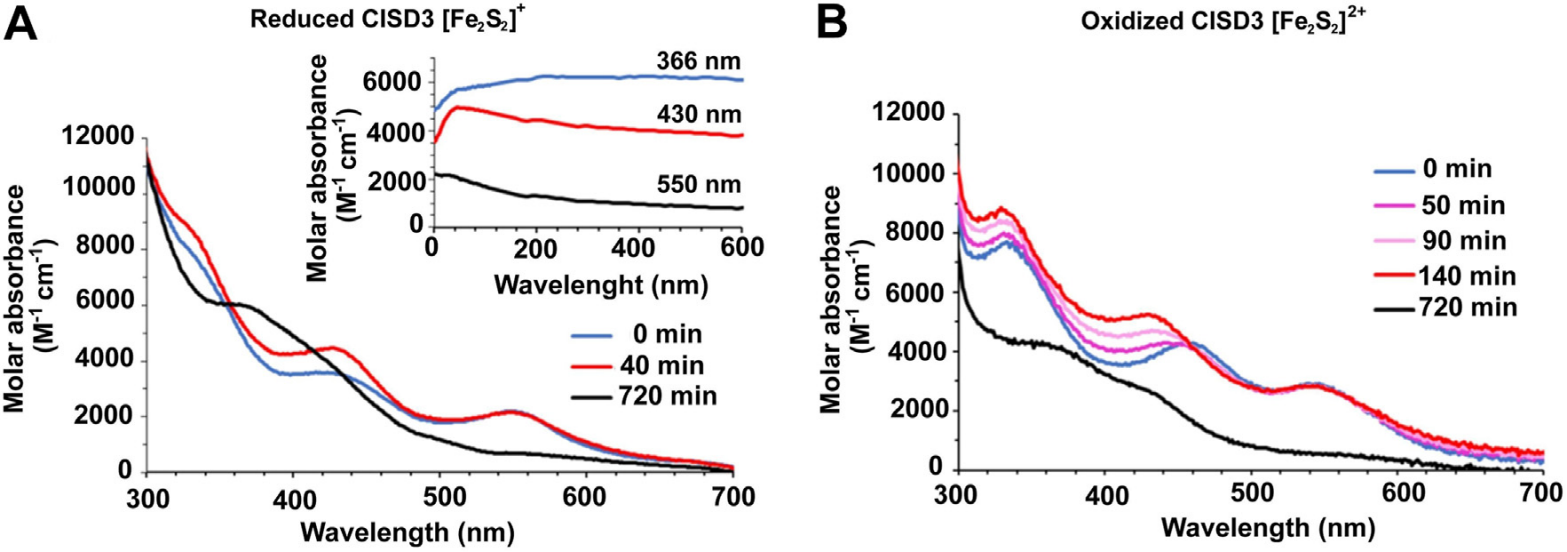
**Interaction of CISD3 with NO followed*****in vitro*****by UV-visible absorption.***A*, UV-visible spectra acquired on reduced CISD3-spermine NONOate mixture under anaerobic conditions at 25 °C: 0 min (*blue*), 40 min (*red*), 720 min (*black*), the inset shows the intensity of the peak at 366 nm (*blue*), at 430 nm (*red*) and at 550 nm (*black*) was as a function of time; *B*, UV-visible spectra acquired on oxidized CISD3-Spermine NONOate mixture under anaerobic conditions at 25 °C: 0 min (*blue*), 50 min (*pink*), 90 min (*light pink*), 140 min (*red*) and 720 min (*black*).

The interaction of CISD3 with NO was followed *in vitro* by UV-visible absorption, NMR and EPR spectroscopies, upon addition of spermine NONOate. Spermine NONOate is a molecule that releases two molecules of NO and has a half-life of 40 min at pH 7.4 and 310 K (50). The UV-visible absorption spectrum, reported in Figure 10*A*, shows that the addition of spermine NONOate to reduced CISD3 under anaerobic conditions gives rise to an intermediate species, monitored by the increase of the absorption bands at 430 nm. When the intensity of this band is plotted *versus* time (Fig. 10*A* inset), a plateau is observed after about 40 min, corresponding to a ratio 1:2 protein:NO. This indicates that NO binds to the reduced clusters in CISD3 as previously proposed (28). When the concentration of NO increases, the UV-visible absorption spectrum evolves. The band at 430 nm decreases, and the band at 550 nm disappears with time, indicating a cluster loss. A new absorption band at 366 nm is observed. The time dependence of the three bands (Fig. 10*A* inset) indicates that the intermediate species observed after 40 min eventually disrupts the [Fe_2_S_2_] cluster, giving rise to a different metal center. The appearance of the 366 nm band has been already observed for Rieske proteins (48) and interpreted as the formation of dinitrosyl iron complexes (DNIC). This behavior is consistent with EPR spectra acquired on reduced CISD3 in the presence of increasing amounts of NO (Fig. S5).

The interaction with NO has also been studied on the oxidized form of CISD3. At variance with the reduced form, UV-visible absorption spectroscopy shows no evidence of a transient intermediate species nor of DNIC complexes. As shown in Figure 10*B*, the band at 460 nm shifts to 430 nm indicating the reduction of the cluster, consistent with the behavior already observed for oxidized CISD3 independently on the addition of NO. Once the cluster is reduced we observe that NO binds to the protein as confirmed by the slightly increase of the band at 430 nm. In about 12 h, the cluster is lost and the DNIC is observed, confirming the behavior of the reduced form.

**Figure 11:**
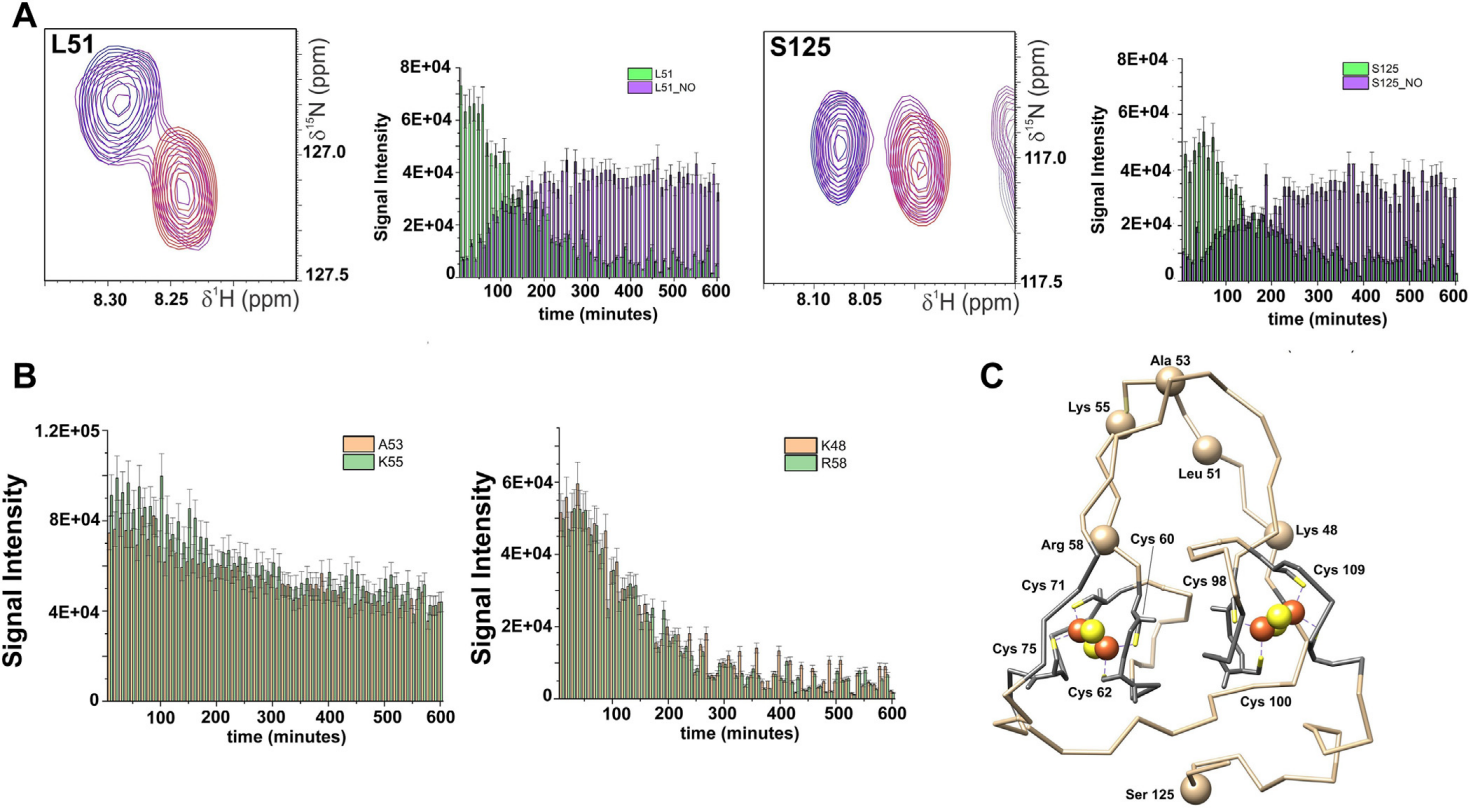
**Stability of reduced CISD3 upon NO treatment monitored by NMR.***A*, representative residues, Leu 51 (*left*) and Ser 125 (*right*) of the slow exchange regime and their respective intensities plotted as graph bars as a function of time. *B*, representative residues, Ala 53 and Lys 55, whose signals intensity slightly decrease as a function of time after the binding of NO (*left*) and the residues (Lys 48 and Arg 58) whose signals dramatically decrease. *C*, representation of the aforementioned residues in the CISD3 structure (PDB code 6AVJ). NO, nitric oxide; PDB, Protein Data Bank.

The addition of spermine NONOate to reduced CISD3 was also followed by ^1^H-^15^N-HSQC spectra. For 1:2 protein:NO ratio, a few residues (Gly 36, Leu 51, Thr 56, Ala 89, Met 94, Gln 119, and Ser 125) showed a second NMR signal that appears in slow exchange with the reduced form, as shown in Figure 11*A* for residues Leu 51 and Ser 125. After about 300 min, the signals of the reduced CISD3 form vanished, and the intensity of the NO bound form is about 65% of the initial intensity, indicating that about 35% of the cluster is lost during the reaction. Consistently, the intensity of ^1^H-^15^N HSQC signals of residues that are not subjected to any change upon NO binding, decrease to ca 65% of their initial intensity, as shown in Figure 11*B* for Ala 53 and Lys 55. Remarkably, residues that are closer to the cluster than Leu 51 and Ser 125, such as Lys 48 and Arg 58 show a different behavior *i.e.*, the disappearance of the NO-free reduced species is 100% and it is not accompanied by the appearance of a NO-bound signal (Fig. 11, *B* and *C*). This can only be due to a longer electronic correlation time of Fe ions in the NO-bound form that causes a larger blind sphere. Signals that in the NO-free reduced CISD3 are affected by paramagnetic relaxation but are still observable are broadened beyond detection in the NO-bound form. This is a very strong evidence that, upon NO binding, the cluster is disassembled but the iron ions are still bound to the protein, thus providing the experimental evidence that the DNIC complex (48, 51) is still bound to the protein, as already proposed for [Fe_4_S_4_] containing proteins (52). In our hands, ^1^H-^15^N HSQC spectra account for a 65% DNIC species, which is higher than 5% previously observed by Cheng *et al* (28) using EPR. However, the quantification of the detected DNIC species is affected by several factors, *i.e.*, NO-donor half-life, [Fe_2_S_2_] clusters and NO concentrations.

In agreement with what was observed by UV-visible absorption spectroscopy, the ^1^H-^15^N HSQC experiments also show that oxidized CISD3 does not bind NO. The protein undergoes reduction and precipitation over time (Fig. S6), as already observed for the oxidized protein without NO.

In conclusion, UV-visible absorption, EPR and NMR spectroscopies provide evidence that NO binds only the reduced form of the [Fe_2_S_2_] clusters of CISD3, forming an intermediate which evolves in thiolate-ligated DNIC species. The blind sphere observed in the NMR spectra of the NO bound species indicates that DNIC species are still bound to iron ions within the protein frame. NO does not interact with oxidized CISD3; instead, the protein undergoes in part cluster loss and reduction of the still bound cluster as observed in the absence of NO.

## Discussion

The *in cellulo* and *in vitro* characterization of human CISD3 provides a better understanding of the mechanisms underlying its function and permits the comparison with the other human NEET proteins, mitoNEET, and CISD2. Our *in cellulo* experiments showed that CISD3 half-life in 50 μM CHX treated cells is about 50 h. In a previous study in HeLa cells (20), mitoNEET half-life was found to be less than 6 h and CISD2 to be highly stable (half-life higher than 48 h), meaning that mitoNEET is particularly unstable compared to the two other members of the NEET family, CISD2 and CISD3.

The high stability of CISD3 in unstressed cells suggests a functional role in fundamental cellular processes, whereas the instability of mitoNEET may indicate that it plays a role in adaptive cellular responses, as already proposed (19). From a structural point of view, NMR data demonstrate that, once the cluster is removed *in vitro*, CISD3 completely loses its 3D structure. The generated apo-form is largely unfolded, unstable, and rapidly precipitates. Consistently, CISD3 levels in iron deficiency conditions (cell treated with DFO) decrease by 50% in about 24 h, reproducing a trend similar to mitoNEET, which was barely detectable after 16 h (20). On the contrary, CISD2 showed no sensitivity to cellular treatment with iron chelators (20). However, the higher cellular instability of CISD3 and mitoNEET under conditions of iron deficiency with respect to CISD2 cannot be explained based on structural properties of their apo-forms *i.e.*, CISD2 stability is potentially a consequence of a greater stability of the apo-CISD2 protein in cells or such different stability is probably linked to their respective functions.

The results obtained by cellular studies raised the question whether CISD3 stability properties are linked to the release of its Fe–S clusters upon cluster oxidation. Protein film voltammetry showed two oxidation states for CISD3: the fully oxidized 2[Fe_2_S_2_]^2+^ and the fully reduced 2[Fe_2_S_2_]^+^ forms. We have observed that the stability of the cluster in oxidized holo-CISD3 in the presence of oxygen is almost independent of the pH, whereas in mitoNEET, it is highly dependent. Oxidized CISD3 loses the clusters also in the presence of H_2_O_2_ under initial anaerobic conditions *in vitro*. However, the pH-dependence of cluster stability in the presence of hydrogen peroxide is not as pronounced as in the case of CISD2 and mitoNEET, which are practically insensitive to hydrogen peroxide at pH 8 (Table 1) (17, 20). Although CISD3 Fe-S clusters are very labile in the presence of H_2_O_2_ *in vitro*, we did not observe a decrease in CISD3 protein levels in H_2_O_2_-treated HEK-293 cells. An explanation could be that CISD3 is protected from the effects of H_2_O_2_ by a partner in the cell, as it has been demonstrated in the case of bacterial nitrogenase and thiol-based peroxidase (53, 54).

3D structures of the NEET proteins may explain the intrinsic pH-independent instability of oxidized CISD3. Although all members of the family share a common structural core, mitoNEET and CISD2 encompass key intersubunit interactions, which are missing in CISD3. In mitoNEET, the interaction between the side chain of Lys 55 and His 87 has been proposed to be the driving factor controlling redox-dependent and pH dependent solvent accessibility and, as a consequence, the overall protein stability (14, 55). In monomeric CISD3, this intersubunit interaction is missing and not replaced; this causes a larger solvent exposure of the reducible iron ion with respect to mitoNEET and gives the molecular basis of the lower and pH independent protein stability of CISD3. In agreement, protein film voltammetry shows that the redox potential remains constant at an average value of ca. −31 mV *versus* SHE with a standard deviation of 6 mV in the pH range 6.5 to 8.3 (Table S1). On the contrary, pronounced pH-dependence has been found for both mitoNEET and CISD2, with redox potential values ranging from about 0 mV at pH 7 to about −50 mV at pH 8.5 (55, 56).

Further insights into the molecular and structural aspects arise from the analysis of NMR data obtained on the two redox states. The distribution of chemical shift perturbations is odd as the largest perturbations are all localized on the β-strands close to the C-term cluster, while corresponding residues at the N-term are affected to a much lesser extent. In metalloproteins, redox shifts can be due either to the change in the electronic structure of the paramagnetic center (57, 58, 59, 60, 61) or to a conformational change associated to the redox process (62, 63). Several studies indicate that redox shifts in Fe-S proteins are not related to the different paramagnetism between oxidized and reduced clusters, but they can be associated with conformational rearrangements (64, 65, 66). In CISD3, a remarkably large redox shift is observed for Lys 48, which is close to the H-bonded residues Ile 47- Leu 97, the latter preceding the iron bound Cys 98 (Fig. 6*B*). Both Ile 47 and Leu 97 are too close to the iron ions to be observed; however, the perturbation observed on Lys 48 suggests that structural changes associated with the redox event may occur at the C-terminal site. Another protein fragment that might be concerned with redox driven structural rearrangements is the terminal α-helix of the CDGSH domain: this helix includes the cluster bound histidine (His 75/His 113) and, from the structural point of view, seems to be important to control the accessibility of the cluster. Also, for this fragment, the large perturbation observed for Ser 115 points out the possibility that structural rearrangements specifically occur at the C-terminal site of the protein (Fig. 6*B*). Remarkably, the amino acids of the C-terminal site of CISD3 are conserved compared to mitoNEET, whereas the α helix at the N-terminal contains two hydrophobic residues (Phe 76 and Phe 77) that are not present in mitoNEET. It seems therefore that when the C_2_ symmetry of mitoNEET (and CISD2) is broken and the two clusters have similar but not identical environments, a conformational change around the C-terminal cluster gives rise to an “open” conformation for the oxidized state. This, redox driven, conformational switch is likely the molecular basis of the protein instability in the oxidized state *i.e.*, the open conformation allows the release of the oxidized clusters. This finding is also completely consistent with the single reduction potential with a cooperative behavior of the two clusters observed by protein film voltammetry. The allosteric communication between the two clusters has been also shown *in silico* in mitoNEET (18). Residues of mitoNEET that are suggested, from a coevolutionary analysis (18), to provide cross talk between the two clusters are retained also in CISD3, explaining the single redox potential observed for two clusters with different environments.

The analysis of the 3D protein structure barely explains that CISD3 protein levels in cells are not affected by H_2_O_2_ whereas they are dramatically affected by NO. In mammals, NO is produced by three different NO synthase enzymes present in a large variety of cells, and a plethora of biological effects have been attributed to NO under physiological or pathophysiological conditions (67, 68). A number of Fe-S cluster-containing proteins in prokaryotes and eukaryotes have been identified both as sensitive sensors of NO and effectors of a cellular adaptive response to damaging NO levels (69, 70, 71). However, only a handful of studies have questioned the reactivity of NEET proteins toward NO and the effects of NO on NEET protein structure, function, stability, and subcellular distribution. Earliest studies have shown no binding of NO to mitoNEET *in vitro* and no mitoNEET protein instability in cells exposed to NO injury. Indeed, Ferecatu *et al.* suggested a protective role for mitoNEET in the repair of NO-mediated damaging of some Fe-S cytosolic proteins such as the iron regulatory protein-1/cytosolic aconitase protein, a central regulator in cellular iron metabolism (19). However, the inability of NO to bind to mitoNEET has been questioned in a more recent study (72) and, to date, mitoNEET sensitivity to NO is still an open question. By contrast, the work of Cheng’s group (28) and this study conclusively demonstrated that NO affects CISD3 in several ways. In cells, we disclosed for the first time a complete degradation of CISD3 induced by prolonged exposure to DETA-NO, a compound mimicking high-throughput NO synthase 2 activity. CISD2 levels were not affected suggesting that NO is not able to interact with this NEET protein, as already proposed *in vitro* (28). Our experiments by UV-visible absorption and EPR spectroscopy provide evidence for NO binding to reduced CISD3, with formation of DNIC, as previously published (28). Likewise, oxidized CISD3 did not react with NO. NMR indicates that [Fe_2_S_2_]^+^ clusters are disrupted in NO-bound CISD3. This might explain the progressive breakdown of CISD3 in cells exposed to NO as the apo-protein is not stable in cells. Our experiments showing that iron deprivation decreases CISD3 stability also support this hypothesis.

NO regulates several important functions and processes in mitochondria, where CISD3 is located. For instance, NO inhibits oxidative phosphorylation and regulates mitochondrial biogenesis and dynamics by different mechanisms including binding to metalloproteins and S-nitrosylation of key proteins (73). Dysregulated production of NO in human tumors has been correlated with the degree of malignancy and poor prognosis for survival (74, 75). NO contributes to metabolic reprogramming in tumors, in particular by stimulating mitochondrial biogenesis. It has been reported that CISD3 expression is largely elevated in various human cancers (22) and it can exert a regulatory role in tumor progression (76). Our study showing that CISD3 is a new target for NO in mitochondria warrants further investigations to determine to what extent NO binding to CISD3 may affect mitochondrial functions, especially in cancer cells exhibiting a dysregulated expression of NO synthase enzymes.

## Experimental procedures

### Cell line, culture, and obtainment of stable CISD3-V5 expressing HEK-293

Human HEK-293 cells were routinely cultured in Dulbecco's modified Eagle's medium containing 4.5 g/L glucose, 2 mM L-glutamine, 10% foetal calf serum and penicillin/streptomycin antibiotics. Cells were transfected using a Lipofectamine 2000 mediated transfection by a pcDNA3.1 plasmid expressing human CISD3 fused to a V5 tag (GKPIPNPLLGLDST) at its C-terminal end. Two days after transfection, cells were selected with G-418 at 1 mg/ml for 2 weeks. The resulting population was tested by immunofluorescence for V5 expression, which was localized exclusively in the mitochondria, and used for further experiments.

### Cell treatments with DFO, SIH, CHX, H_2_O_2,_ and DETA-NO

Stable CISD3-V5 expressing HEK-293 cells were seeded at 0.34 × 10^6^ cells/cm^2^, incubated with either DFO or SIH, or 50 μM of CHX for different times. Cell treatment with a protein synthesis inhibitor such as CHX is widely used to determine the half-life of a given protein (77). CHX inhibits translational elongation by interfering with the translocation step in protein synthesis. Treatment of the cells with CHX in a time-dependent decay experiment followed by Western blotting of the cell lysates can provide an estimation of the protein half time. For NO and H_2_O_2_ challenge studies, CISD3-V5 overexpressing HEK-293 cells were seeded the day before and then exposed or not to DETA-NO or H_2_O_2_. The treatment was performed at increasing concentrations (0, 25, 50, 100, and 200 μM) for H_2_O_2_ and (0, 62,5, 125, 250, and 500 μM) for DETA-NONOate. Then we selected 100 μM and 250 μM concentrations for H_2_O_2_ and DETA-NONOate, respectively, to evaluate the half-life of CISD3-V5, quantifying protein amount at 0, 1, 5, 11, and 24 h. Autophagy inhibitors (3-methyladenine and chloroquine) were used with and without DFO 50 μM at 5 mM and 50 μM, respectively. Chemicals except DETA-NO (Cayman Chemical) were from Merck.

### Immunoblot analysis of cell extracts

Total protein extracts from HEK-293 cells were obtained by harvesting cells in Laemmli buffer (0.06 M Tris–HCl pH 6.8, 10% glycerol, and 2% SDS) containing protease inhibitors (Sigma-Aldrich # P8340) and 2 mM Pefabloc (Calbiochem). Protein concentrations were determined using the detergent compatible protein assay (Bio-Rad). Equal amounts (30 μg) of proteins were separated on a 16% acrylamide SDS–PAGE and transferred on 0.45 μm of polyvinylidene difluoride membranes. The primary antibodies used were: anti-α-tubulin (Sigma–Aldrich #A5441) 1:1000, anti-CISD2 (Proteintech #13318-1-AP) 1:1000, anti-GADPH (Novus Biologicals #NBP2-37563) 1:500, and anti-V5 (Novus Biologicals # NB600-381) 1:2000. Secondary antibodies were Alexa Fluor680–conjugated goat anti-mouse IgGs (A-21057, Life Technologies) 1:10,000 and IRDye 800CW–conjugated goat anti-rabbit IgGs (926-32211, Li-Cor Biosciences) 1:15,000.

### Purification of holo-CISD3 for *in vitro* studies

The CISD3 ORF without the mitochondrial targeting signal peptide (amino acids 37–127) was inserted into a pET28a(+) plasmid. The plasmid was used to transform *E. coli* BL21 (DE3) GOLD competent cells. Cell growth was performed at 37 °C in either LB, for nonlabeled protein, or in M9 minimal media supplemented with 1.2 g/L of (^15^NH_4_)_2_SO_4_, 3 g/L of glucose, 4 ml of Q solution (metal mix solution: 50 mM FeCl_3_, 20 mM CaCl_2_, 10 mM MnCl_2_, 10 mM ZnSO_4_, 2 mM of each of CoCl_2_, CuCl_2_, NiCl_2_, Na_2_MnO_4_, Na_2_SeO_3_, and H_3_BO_3_). A total of 500 μM Mohr’s Salt was added when the cell culture reached an *A*_600_ of 0.6. At an *A*_600_ of 0.8 to 1, the temperature was lowered to 18 °C and the protein overexpression was induced with 0.1 mM IPTG. Cells were incubated at 18 °C overnight and centrifuged.

Cells were resuspended in 80 ml of 20 mM Tris–HCl pH 7.5 buffer and lysed by adding CelLytic Reagent (0.8 g/1 L culture) (Merck). After centrifugation, the supernatant was loaded on a 5 ml HiTrap SP FF cationic exchange column. The column was washed with a linear NaCl gradient (0–500 mM) until CISD3 protein solution eluted with 20 mM Tris–HCl pH 7.5300 mM NaCl. The fractions containing the protein were collected, concentrated, and injected in Superdex 200 Increase 16/60 75 pg previously equilibrated with Tris–HCl 50 mM NaCl 150 mM pH 7.5 buffer. All the buffers used for purification were rendered oxygen-depleted by introducing a nitrogen flux into the solution. The iron content of the reduced CISD3, determined following a procedure reported in the articles (78, 79, 80, 81) is consistent with an 85% metallated protein (∼1.70 [Fe_2_S_2_]^2+^ clusters per CISD3 protein).

### *In vitro* CISD3 cluster loss

UV-visible absorption spectroscopy was used to evaluate the kinetic of cluster loss of CISD3 cluster in both redox states, under aerobic and anaerobic conditions and at different pH. The purification of CISD3 was carried out in an aerobic environment, following the same protocol described above for the anaerobic purification. This ensures that the initial redox state of CISD3 is oxidized. After purification, the protein was concentrated and then put in an anaerobic environment. Cluster loss reactions were performed in degassed buffer (100 mM Bis–Tris 100 mM NaCl for pH 5.8, 6.3 and pH 7 or 100 mM Tris–HCl 100 mM NaCl for pH 8). Reduced CISD3 was obtained by addition of 20 mM sodium dithionite. The anaerobic samples were sealed under an anaerobic atmosphere, whereas the aerobic samples were exposed to the oxygen in the atmosphere. Cluster loss reactions were followed by the acquisition of (240–900 nm) UV-visible absorption spectra over time by Cary 100 (Agilent Technologies) spectrophotometer equipped with a temperature control apparatus set at 25 °C. Cluster loss was measured for each time t using R(t) = (A_(t)_-A_initial_)/(A_final_-A_initial_) with R_(t)_, the reaction progress at time t, A the absorbance at 460 nm for the oxidized cluster and 550 nm for the reduced cluster and A_initial_ and A_final_, the absorbance at the beginning and at the end (when the cluster loss is complete) of the reaction, respectively.

### NMR spectroscopy

A combination of NMR experiments was either recorded on Bruker AVANCE 500 MHz, 900 MHz, and 950 MHz spectrometers, on 0.3 to 0.5 mM ^15^N-labeled samples in 20 mM Tris–HCl buffer, 150 mM NaCl, pH 7.5, 10% (v/v) D_2_O, at 298 K. All NMR spectra were collected and processed using Bruker (www.bruker.com) software (Topspin 3.6). ^1^H-^15^N HSQC spectra were recorded on purified CISD3 protein in its reduced 2[Fe_2_S_2_]^+^ and oxidized 2[Fe_2_S_2_]^2+^ forms with the following parameters: 56 ms and 31 ms for direct (t_2_max) and indirect (t_1_max) acquisition times and a recycle delay of 1 s. A data-matrix of 1024 × 128 complex points, with eight transients each increment, was collected. The auto-reduction process of 2[Fe_2_S_2_]^2+^ CISD3 was monitored recording ^1^H-^15^N HSQC experiments at 298 K on a 900 MHz spectrometer along a period of 10 h, with the parameter set described above. The paramagnetism tailored ^1^H-^15^N HSQC-AP experiments (82) were recorded using the following parameters: 27 ms and 31 ms for direct (t_2_max) and indirect (t_1_max) acquisition times and a recycle delay of 150 ms. A data-matrix of 1024 × 128 complex points, with 128 transients each increment, was collected. The interaction between NO and both oxidized and reduced forms of CISD3 was monitored at 11.7 and 21.1 T fields at 298 K, using a standard SOFAST-HMQC pulse sequence with the following parameters: 35 ms and 20 ms for direct (t_2_max) and indirect (t_1_max) acquisition times and a recycle delay of 200 ms. A data-matrix of 1024 × 128 complex points, with 16 transients each increment, was collected (83). ^15^N-labeled samples were prepared to a final concentration of 0.5 mM (20 mM Tris–HCl buffer, 150 mM NaCl, pH 7.5, and 10% (v/v) D_2_O), and spermine NONOate was added up to a 1:2 protein:NO ratio. The NMR titration with H_2_O_2_ was performed on 0.6 mM ^15^N-labeled CISD3 protein samples by adding 2 equivalents and 4 equivalents of H_2_O_2_, to a final concentration of 2.4 mM.

### Protein film voltammetry

Protein film voltammetry was performed at 25 °C inside an anaerobic glove box (Coy) using a double-walled vessel thermostated using an external water bath. A potentiostat (CH Instruments 1201A) was connected to a pyrolitic graphite electrode (PGE) working electrode, a graphite counter electrode, and an Ag/AgCl reference electrode (CH Instruments). The working electrode was treated for 5 min in 1 M nitric acid and thoroughly washed with double distilled water. It was then polished with Alumina (1 μm and 0.3 μm) washed thoroughly with double distilled water, and left to dry inside the glove box. Ten microliters of the 650 μM protein solution was deposited in the electrode and left to dry for at least 1 h. The electrode was placed in the buffer solution (50 mM phosphate equilibrated at various pH values from 6.5 to 8.2) and three complete scans were collected at a scan rate of 50 and 100 mV/s. The pH of the solutions was checked after each experiment. Raw voltammograms were processed with SOAS (84) to subtract the capacitive current and the position of the anodic and cathodic peaks was averaged and is reported *versus* SHE by adding 210 mV to the value obtained *versus* Ag/AgCl.

### EPR spectroscopy

EPR spectra were acquired using a Spectrometer ELEXSYS E580 operating with at 9.8 GHz continuous wave X-band, with a power level of 0.1 mW, 100 kHz modulation frequency, 1.2 mT amplitude modulation, centered at 350 mT with a field sweep of 200 mT and at 20 K.

#### In-cell sample preparation

*E. coli* BL21 (DE3) GOLD were transformed with the pET28a(+) plasmid containing CISD3 gene (amino acids 37–127). A 5 ml-preculture in LB medium was incubated overnight at 37 °C. One microliter of this preculture was inoculated into 100 ml of LB, and the cells were grown at 37 °C up to *A*_600_ of 0.8. Subsequently, protein overexpression was induced overnight at 18 °C by addition of 0.1 mM IPTG. The cells were pelleted by centrifugation and washed twice with M9 medium to remove the culture medium containing waste products. The blank was performed following the same procedure with the only exception that protein overexpression was not induced. Sample cell numbers were normalized by measuring the *A*_600_. Cells were resuspended in 300 μl of PBS containing 20% glucose and placed in the EPR tube which was frozen in liquid nitrogen.

#### In-vitro sample preparation

Protein samples were prepared in a buffered solution containing 50 mM phosphate, 150 mM NaCl, pH 7.5 at a concentration of 40 μM. To monitor the interaction of CISD3 protein with NO we added one or two equivalents of spermine-NONOate in a stock solution and let it react up to 24 h. At 5, 210, and 1440 min from the addition of the molecule, 200 μl were collected in an EPR tube for data acquisition.

## Data availability

All the data described in the manuscript are contained within it.

## Supporting information

This article contains supporting information.

## Conflict of interest

The authors declare that they have no conflicts of interest with the contents of this article.

## Supporting information

Supporting Table S1

Supporting Informaton

## References

[1] Tamir S., Paddock M.L., Darash-Yahana-Baram M., Holt S.H., Sohn Y.S., Agranat L. (2015). Structure-function analysis of NEET proteins uncovers their role as key regulators of iron and ROS homeostasis in health and disease. Biochim. Biophys. Acta.

[2] Wiley S.E., Murphy A.N., Ross S.A., van der Geer P., Dixon J.E. (2007). MitoNEET is an iron-containing outer mitochondrial membrane protein that regulates oxidative capacity. Proc. Natl. Acad. Sci. U. S. A..

[3] Wiley S.E., Paddock M.L., Abresch E.C., Gross L., van der Geer P., Nechushtai R. (2007). The outer mitochondrial membrane protein mitoNEET contains a novel redox-active 2Fe-2S cluster. J. Biol. Chem..

[4] Valasatava Y., Rosato A., Banci L., Andreini C. (2016). Metalpredator: a web server to predict iron-sulfur cluster binding proteomes. Bioinformatics.

[5] Andreini C., Putignano V., Rosato A., Banci L. (2018). The human iron-proteome. Metallomics.

[6] Colca J.R., McDonald W.G., Waldon D.J., Leone J.W., Lull J.M., Bannow C.A. (2004). Identification of a novel mitochondrial protein ("mitoNEET") cross-linked specifically by a thiazolidinedione photoprobe. Am. J. Physiol. Endocrinol. Metab..

[7] Song G., Tian F., Liu H., Li G., Zheng P. (2021). Pioglitazone inhibits metal cluster transfer of mitoNEET by Stabilizing the labile Fe-N bond revealed at single-bond level. J. Phys. Chem. Lett..

[8] Takahashi T., Yamamoto M., Amikura K., Kato K., Serizawa T., Serizawa K. (2015). A novel MitoNEET ligand, TT01001, improves diabetes and ameliorates mitochondrial function in db/db mice. J. Pharmacol. Exp. Ther..

[9] Inupakutika M.A., Sengupta S., Nechushtai R., Jennings P.A., Onuchic J.N., Azad R.K. (2017). Phylogenetic analysis of eukaryotic NEET proteins uncovers a link between a key gene duplication event and the evolution of vertebrates. Sci. Rep..

[10] Lipper C.H., Karmi O., Sohn Y.S., Darash-Yahana M., Lammert H., Song L. (2018). Structure of the human monomeric NEET protein MiNT and its role in regulating iron and reactive oxygen species in cancer cells. Proc. Natl. Acad. Sci. U. S. A..

[11] Mittler R., Darash-Yahana M., Sohn Y.S., Bai F., Song L., Cabantchik I.Z. (2019). NEET proteins: a new link between iron metabolism, reactive oxygen species, and cancer. Antioxid. Redox Signal..

[12] Karmi O., Marjault H.B., Pesce L., Carloni P., Onuchic J.N., Jennings P.A. (2018). The unique fold and lability of the [2Fe-2S] clusters of NEET proteins mediate their key functions in health and disease. J. Biol. Inorg. Chem..

[13] Valer L., Rossetto D., Parkkila T., Sebastianelli L., Guella G., Hendricks A.L. (2022). Histidine ligated iron-sulfur peptides. Chembiochem.

[14] Gee L.B., Pelmenschikov V., Mons C., Mishra N., Wang H., Yoda Y. (2021). NRVS and DFT of MitoNEET: understanding the special Vibrational structure of a [2Fe-2S] cluster with (Cys)(3)(His)(1) ligation. Biochemistry.

[15] Karmi O., Marjault H.B., Bai F., Roy S., Sohn Y.S., Darash Yahana M. (2022). A VDAC1-mediated NEET protein chain transfers [2Fe-2S] clusters between the mitochondria and the cytosol and impacts mitochondrial dynamics. Proc. Natl. Acad. Sci. U. S. A..

[16] Golinelli-Cohen M.P., Lescop E., Mons C., Goncalves S., Clemancey M., Santolini J. (2016). Redox control of the human iron-sulfur repair protein MitoNEET activity via its iron-sulfur cluster. J. Biol. Chem..

[17] Mons C., Botzanowski T., Nikolaev A., Hellwig P., Cianferani S., Lescop E. (2018). The H2O2-resistant Fe-S redox switch MitoNEET Acts as a pH sensor to repair stress-Damaged Fe-S protein. Biochemistry.

[18] Zuo K., Marjault H.-B., Bren K.L., Rossetti G., Nechushtai R., Carloni P. (2021). The two redox states of the human NEET proteins’ [2Fe–2S] clusters. J. Biol. Inorg. Chem..

[19] Ferecatu I., Goncalves S., Golinelli-Cohen M.P., Clemancey M., Martelli A., Riquier S. (2014). The diabetes drug target MitoNEET governs a novel trafficking pathway to rebuild an Fe-S cluster into cytosolic aconitase/iron regulatory protein 1. J. Biol. Chem..

[20] Salameh M., Riquier S., Guittet O., Huang M.E., Vernis L., Lepoivre M. (2021). New insights of the NEET protein CISD2 reveals distinct features compared to its close mitochondrial homolog mitoNEET. Biomedicines.

[21] Pérez-Ramírez M., Hernández-Jiménez A.J., Guerrero-Guerrero A., Benadón-Darszon E., Pérezpeña-Díazconti M., Siordia-Reyes A.G. (2016). Genomics and epigenetics: a study of ependymomas in pediatric patients. Clin. Neurol. Neurosurg..

[22] Li Y., Wang X., Huang Z., Zhou Y., Xia J., Hu W. (2021). CISD3 inhibition drives cystine-deprivation induced ferroptosis. Cell Death Dis..

[23] Zhang C., Liu X., Jin S., Chen Y., Guo R. (2022). Ferroptosis in cancer therapy: a novel approach to reversing drug resistance. Mol. Cancer.

[24] Fanzani A., Poli M. (2017). Iron, oxidative damage and ferroptosis in Rhabdomyosarcoma. Int. J. Mol. Sci..

[25] King S.D., Gray C.F., Song L., Mittler R., Padilla P.A. (2021). The mitochondrial localized CISD-3.1/CISD-3.2 proteins are required to maintain normal germline structure and function in Caenorhabditis elegans. PLoS One.

[26] Marjault H.B., Karmi O., Rowland L., Nguyen T.T., Grant D., Manrique-Acevedo C. (2023). CISD3 is required for Complex I function, mitochondrial integrity, and skeletal muscle maintenance. bioRxiv.

[27] Gashaw I., Ellinghaus P., Sommer A., Asadullah K. (2011). What makes a good drug target?. Drug Discov. Today.

[28] Cheng Z., Landry A.P., Wang Y., Ding H. (2017). Binding of nitric oxide in CDGSH-type [2Fe-2S] clusters of the human mitochondrial protein Miner2. J. Biol. Chem..

[29] Infante T., Costa D., Napoli C. (2021). Novel insights regarding nitric oxide and cardiovascular diseases. Angiology.

[30] Ghasemi M., Mayasi Y., Hannoun A., Eslami S.M., Carandang R. (2018). Nitric oxide and mitochondrial function in Neurological diseases. Neuroscience.

[31] Guzik T.J., Korbut R., Adamek-Guzik T. (2003). Nitric oxide and superoxide in inflammation and immune regulation. J. Physiol. Pharmacol..

[32] Ghimire K., Altmann H.M., Straub A.C., Isenberg J.S. (2017). Nitric oxide: what's new to NO?. Am. J. Physiol. Cell Physiol..

[33] Tuteja N., Chandra M., Tuteja R., Misra M.K. (2004). Nitric oxide as a unique bioactive signaling Messenger in physiology and pathophysiology. J. Biomed. Biotechnol..

[34] Hirst D.G., Robson T., McCarthy H.O., Coulter J.A. (2011). Nitric Oxide: Methods and Protocols.

[35] Grifagni D., Silva J.M., Cantini F., Piccioli M., Banci L. (2023). Relaxation-based NMR assignment: spotlights on ligand binding sites in human CISD3. J. Inorg. Biochem..

[36] Silva J.M., Grifagni D., Cantini F., Piccioli M. (2023). (1)H, (13)C and (15)N assignment of the human mitochondrial paramagnetic iron-sulfur protein CISD3. Biomol. NMR Assign..

[37] Hara Y., Yanatori I., Tanaka A., Kishi F., Lemasters J.J., Nishina S. (2020). Iron loss triggers mitophagy through induction of mitochondrial ferritin. EMBO Rep..

[38] Yang Y.P., Hu L.F., Zheng H.F., Mao C.J., Hu W.D., Xiong K.P. (2013). Application and interpretation of current autophagy inhibitors and activators. Acta pharmacol. Sin..

[39] Jeuken L.J.C., Jones A.K., Chapman S.K., Cecchini G., Armstrong F.A. (2002). Electron-transfer mechanisms through biological redox chains in multicenter enzymes. J. Am. Chem. Soc..

[40] Léger C., Elliott S.J., Hoke K.R., Jeuken L.J., Jones A.K., Armstrong F.A. (2003). Enzyme electrokinetics: using protein film voltammetry to investigate redox enzymes and their mechanisms. Biochemistry.

[41] Dalgleish D.G. (1981). Biophysical Chemistry: Part III 'The Behaviour of Biological Macromolecules: by CR Cantor and PR Schimmel. With two Appendices and Index to Parts I-III. pp 849-1371. WH Freeman, Oxford. 1980. £22.40 and £11.90 (paperback). Biochem. Educ..

[42] Mons C., Ferecatu I., Riquier S., Lescop E., Bouton C., Golinelli-Cohen M.-P., David S.S. (2017).

[43] Camponeschi F., Gallo A., Piccioli M., Banci L. (2021). The long-standing relationship between Paramagnetic NMR and Iron-Sulfur proteins: the mitoNEET example. An old method for new stories or the other way around?. Magn. Reson.(Gott).

[44] Trindade I.B., Coelho A., Cantini F., Piccioli M., Louro R.O. (2022). NMR of paramagnetic metalloproteins in solution: Ubi venire, quo vadis?. J. Inorg. Biochem..

[45] Trindade I.B., Invernici M., Cantini F., Louro R.O., Piccioli M. (2021). Sequence-specific assignments in NMR spectra of paramagnetic systems: a non-systematic approach. Inorg. Chim. Acta.

[46] Banci L., Camponeschi F., Ciofi-Baffoni S., Piccioli M. (2018). Correction to: the NMR contribution to protein-protein networking in Fe-S protein maturation. J. Biol. Inorg. Chem..

[47] Hsueh K.L., Westler W.M., Markley J.L. (2010). NMR investigations of the Rieske protein from Thermus thermophilus support a coupled proton and electron transfer mechanism. J. Am. Chem. Soc..

[48] Tinberg C.E., Tonzetich Z.J., Wang H., Do L.H., Yoda Y., Cramer S.P. (2010). Characterization of iron dinitrosyl species formed in the reaction of nitric oxide with a biological Rieske center. J. Am. Chem. Soc..

[49] Landry A.P., Ding H. (2014). Redox control of human mitochondrial outer membrane protein MitoNEET [2Fe-2S] clusters by biological thiols and hydrogen peroxide. J. Biol. Chem..

[50] Moncada S., Palmer R.M., Higgs E.A. (1991). Nitric oxide: physiology, pathophysiology, and pharmacology. Pharmacol. Rev..

[51] Butler A.R., Megson I.L. (2002). Non-heme iron nitrosyls in biology. Chem. Rev..

[52] Foster M.W., Cowan J.A. (1999). Chemistry of nitric oxide with protein-bound iron sulfur centers. Insights on physiological reactivity. J. Am. Chem. Soc..

[53] Schlesier J., Rohde M., Gerhardt S., Einsle O. (2016). A conformational switch triggers nitrogenase protection from oxygen damage by Shethna protein II (FeSII). J. Am. Chem. Soc..

[54] Fourquet S., Huang M.E., D'Autreaux B., Toledano M.B. (2008). The dual functions of thiol-based peroxidases in H2O2 scavenging and signaling. Antioxid. Redox Signal..

[55] Bak D.W., Zuris J.A., Paddock M.L., Jennings P.A., Elliott S.J. (2009). Redox characterization of the FeS protein MitoNEET and impact of thiazolidinedione drug binding. Biochemistry.

[56] Conlan A.R., Axelrod H.L., Cohen A.E., Abresch E.C., Zuris J., Yee D. (2009). Crystal structure of Miner1: the redox-active 2Fe-2S protein causative in Wolfram Syndrome 2. J. Mol. Biol..

[57] Miao Q., Nitsche C., Orton H., Overhand M., Otting G., Ubbink M. (2022). Paramagnetic chemical Probes for studying biological Macromolecules. Chem. Rev..

[58] Welegedara A.P., Yang Y., Lee M.D., Swarbrick J.D., Huber T., Graham B. (2017). Double-arm Lanthanide tags deliver narrow Gd3+-Gd3+ distance distributions in double electron-electron resonance (DEER) measurements. Chemistry.

[59] Nitsche C., Otting G. (2017). Pseudocontact shifts in biomolecular NMR using paramagnetic metal tags. Prog. Nucl. Magn. Reson. Spectrosc..

[60] Bertarello A., Benda L., Sanders K., Pell A.J., Knight M.J., Pelmenschikov V. (2020). Pico-meter resolution structure of the coordination sphere in the metal-binding site in a metalloprotein by NMR. J. Am. Chem. Soc..

[61] Cerofolini L., Silva J.M., Ravera E., Romanelli M., Geraldes C., Macedo A.L. (2019). How do nuclei Couple to the Magnetic Moment of a paramagnetic center? A new theory at the Gauntlet of the experiments. J. Phys. Chem. Lett..

[62] Messias A.C., Kastrau D.H.W., Costa H.S., LeGall J., Turner D.L., Santos H. (1998). Solution structure of Desulfovibrio vulgaris (Hildenborough) ferrocytochrome c3: structural basis for functional cooperativity. J. Mol. Biol..

[63] Paquete C.M., Saraiva I.H., Calçada E., Louro R.O. (2010). Molecular basis for directional electron transfer. J. Biol. Chem..

[64] Lehmann T.E., Luchinat C., Piccioli M. (2002). Redox-related chemical shift perturbations on backbone nuclei of high-potential iron sulfur proteins. Inorg. Chem..

[65] Pochapsky T.C., Kostic M., Jain N., Pejchal R. (2001). Redox-dependent conformational selection in a Cys_4_Fe_2_S_2_ ferredoxin. Biochemistry.

[66] Xia B., Volkman B.F., Markley J.L. (1998). Evidence for oxidation-state-dependent conformational changes in human ferredoxin from multinuclear, multidimensional NMR spectroscopy. Biochemistry.

[67] Clementi E., Brown G.C., Feelisch M., Moncada S. (1998). Persistent inhibition of cell respiration by nitric oxide: crucial role of S-nitrosylation of mitochondrial complex I and protective action of glutathione. Proc. Natl. Acad. Sci. U. S. A..

[68] Drapier J.C. (1997). Interplay between NO and [Fe-S] clusters: relevance to biological systems. Methods.

[69] Mettert E.L., Kiley P.J. (2015). Fe-S proteins that regulate gene expression. Biochim. Biophys. Acta.

[70] Vine C.E., Cole J.A. (2011). Nitrosative stress in Escherichia coli: reduction of nitric oxide. Biochem. Soc. Trans..

[71] Spiro S. (2007). Regulators of bacterial responses to nitric oxide. FEMS Microbiol. Rev..

[72] Fontenot C.R., Cheng Z., Ding H. (2022). Nitric oxide reversibly binds the reduced [2Fe-2S] cluster in mitochondrial outer membrane protein mitoNEET and inhibits its electron transfer activity. Front. Mol. Biosci..

[73] López-Sánchez L.M., Aranda E., Rodríguez-Ariza A. (2020). Nitric oxide and tumor metabolic reprogramming. Biochem. Pharmacol..

[74] Ekmekcioglu S., Ellerhorst J.A., Prieto V.G., Johnson M.M., Broemeling L.D., Grimm E.A. (2006). Tumor iNOS predicts poor survival for stage III melanoma patients. Int. J. Cancer.

[75] Ko E.J., Kim E.J., Cho H.J., Oh J., Park H.S., Ryu C.S. (2022). Prognostic significance of three endothelial nitric oxide synthase (eNOS) polymorphisms and metabolic syndrome (MetS) in patients with colorectal cancer. Genes Genomics.

[76] Jiang H., Fang Y., Wang Y., Li T., Lin H., Lin J. (2023). FGF4 improves hepatocytes ferroptosis in autoimmune hepatitis mice via activation of CISD3. Int. Immunopharmacol..

[77] Kao S.H., Wang W.L., Chen C.Y., Chang Y.L., Wu Y.Y., Wang Y.T. (2015). Analysis of protein stability by the cycloheximide Chase assay. Bio Protoc..

[78] Nechushtai R., Conlan A.R., Harir Y., Song L., Yogev O., Eisenberg-Domovich Y. (2012). Characterization of Arabidopsis NEET reveals an ancient role for NEET proteins in iron metabolism. Plant Cell.

[79] Lipper C.H., Paddock M.L., Onuchic J.N., Mittler R., Nechushtai R., Jennings P.A. (2015). Cancer-related NEET proteins transfer 2Fe-2S clusters to Anamorsin, a protein required for cytosolic iron-sulfur cluster biogenesis. PLoS One.

[80] Banci L., Bertini I., Ciofi-Baffoni S., Boscaro F., Chatzi A., Mikolajczyk M. (2011). Anamorsin is a 2Fe2S cluster-containing substrate of the Mia40-dependent mitochondrial protein trapping machinery. Chem. Biol..

[81] Maione V., Grifagni D., Torricella F., Cantini F., Banci L. (2020). CIAO3 protein forms a stable ternary complex with two key players of the human cytosolic iron-sulfur cluster assembly machinery. J. Biol. Inorg. Chem..

[82] Ciofi-Baffoni S., Gallo A., Muzzioli R., Piccioli M. (2014). The IR-(1)(5)N-HSQC-AP experiment: a new tool for NMR spectroscopy of paramagnetic molecules. J. Biomol. NMR.

[83] Schanda P., Kupce E., Brutscher B. (2005). SOFAST-HMQC experiments for recording two-dimensional heteronuclear correlation spectra of proteins within a few seconds. J. Biomol. NMR.

[84] Fourmond V., Hoke K., Heering H.A., Baffert C., Leroux F., Bertrand P. (2009). SOAS: a free program to analyze electrochemical data and other one-dimensional signals. Bioelectrochemistry.

