# Supplementary material for: Biochemical and cellular characterization of the CISD3 protein: Molecular bases of cluster release and destabilizing effects of nitric oxide
Source: J Biol Chem. 2024 Feb 12;300(3):105745. doi: 10.1016/j.jbc.2024.105745 (PMC10937110; doi:10.1016/j.jbc.2024.105745)
Supplement: Supporting Table S1 [file mmc1.docx]

**Table S1:** Potential vs SHE of CISD3 at different pH.

| pH | Potential (V) vs SHE |
| --- | --- |
| 6.46 | -0.029 |
| 6.49 | -0.027 |
| 6.63 | -0.029 |
| 7.8 | -0.033 |
| 8.26 | -0.033 |
