# Supplementary material for: Biochemical and cellular characterization of the CISD3 protein: Molecular bases of cluster release and destabilizing effects of nitric oxide
Source: J Biol Chem. 2024 Feb 12;300(3):105745. doi: 10.1016/j.jbc.2024.105745 (PMC10937110; doi:10.1016/j.jbc.2024.105745)
Supplement: Supporting Informaton [file mmc2.pdf]

Biochemical and cellular characterization of the CISD3 protein: molecular bases of cluster release and destabilizing effects of nitric oxide

Deborah Grifagni[a] , José Malanho Silva[a], Leonardo Querci[a], Michel Lepoivre[b], Cindy Vallières[b], Ricardo O Louro[c], Lucia Banci[a], Mario Piccioli[a*], Marie-Pierre Golinelli-Cohen[b*], Francesca Cantini[a*]

[a] Magnetic Resonance Center and Department of Chemistry, University of Florence, Via L. Sacconi 6 50019 Sesto Fiorentino, Italy.

[b] Université Paris-Saclay, CNRS, Institut de Chimie des Substances Naturelles, UPR 2301, 91198 Gif-sur-Yvette, France.

[c] Instituto de Tecnologia Química e Biológica António Xavier (ITQB-NOVA), Universidade Nova de Lisboa, Av. da República (EAN), 2780-157 Oeiras, Portugal.

Supplementary Materials

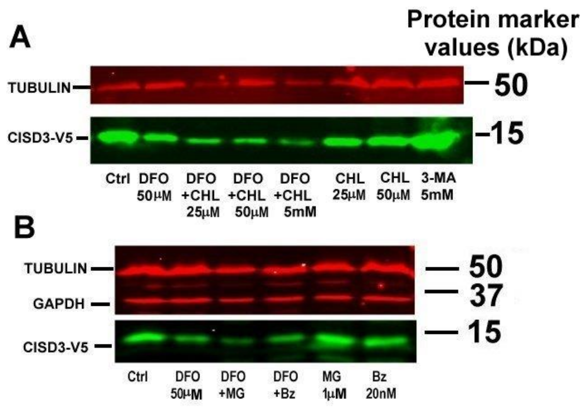

Figure S1. Autophagy and proteasome inhibitors fail to prevent decrease in CISD3-V5 expression induced by iron chelators. Immunoblot of HEK-293 cells overexpressing CISD3-V5 incubated for 24h with 50 µM DFO in the presence or the absence of a) the autophagy inhibitors 3-methyladenine (3-MA, 5mM) and chloroquine (CHL, 25 and 50 µM)., and b) the proteasome inhibitors MG-132 (MG, 1 µM) and bortezomib (Bz, 20 nM). Tubulin and GAPDH were used as loading controls. The ladder of the protein marker is reported on the right.

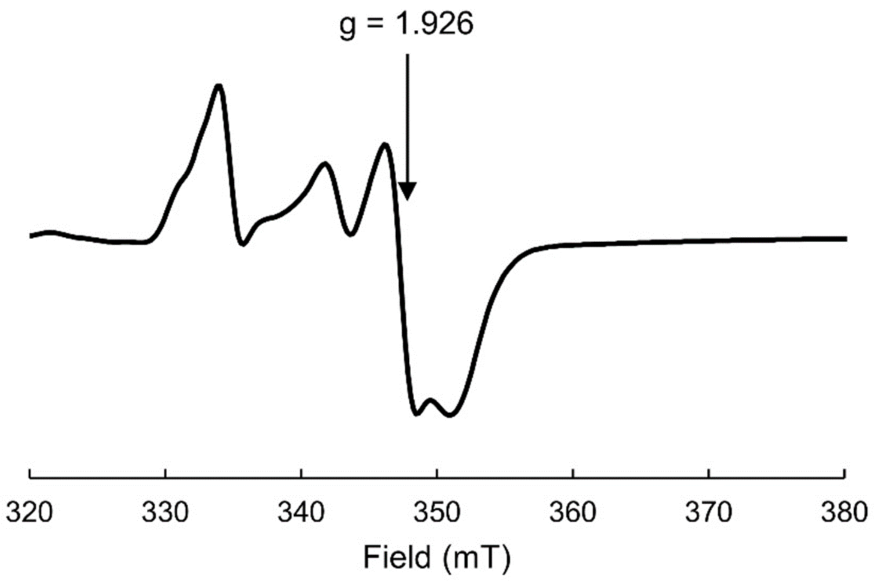

Figure S2: In-cellulo EPR spectrum of CISD3 in *E. coli*.

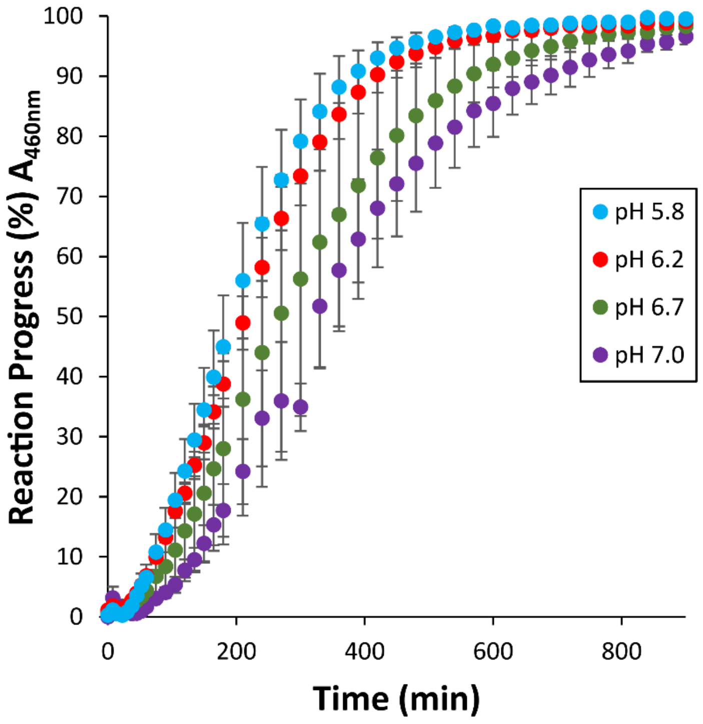

Figure S3: Cluster loss reactions of the oxidized cluster under aerobic conditions. The intensity of the peak at 460 nm was monitored as a function of the time. Curves describing the pH dependence under aerobic conditions at 25°C followed by UV-visible absorption spectroscopy of $[2Fe\text{-}2S]^{2+}$ CISD3, pH 5.8 (blue), 6.2 (red), 6.7 (green) and 7.0 (purple)

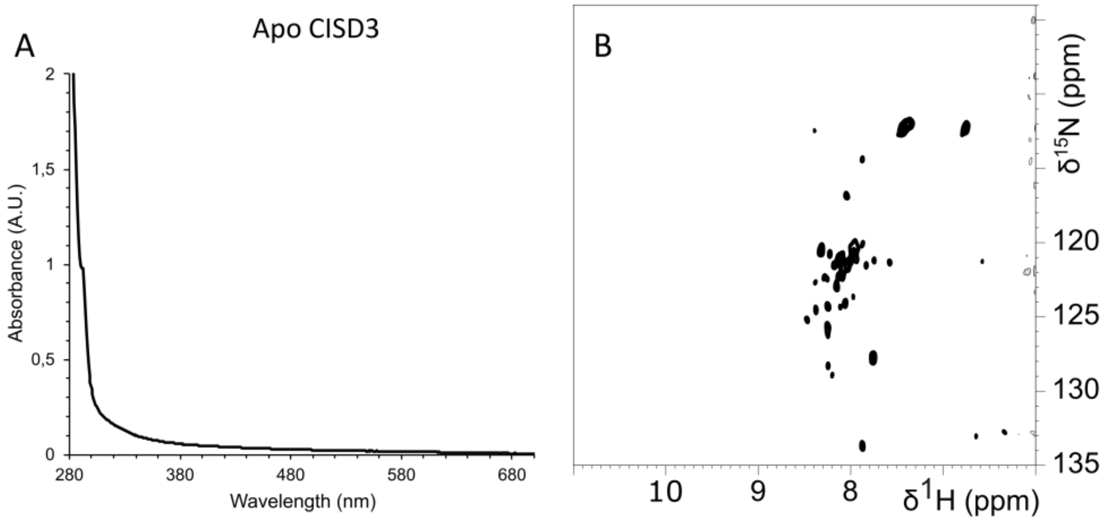

Figure S4: Spectroscopic characterization of CISD3 following treatment with EDTA: (a) UV-vis absorption spectrum and (b) ^{15}N HSQC NMR spectrum.

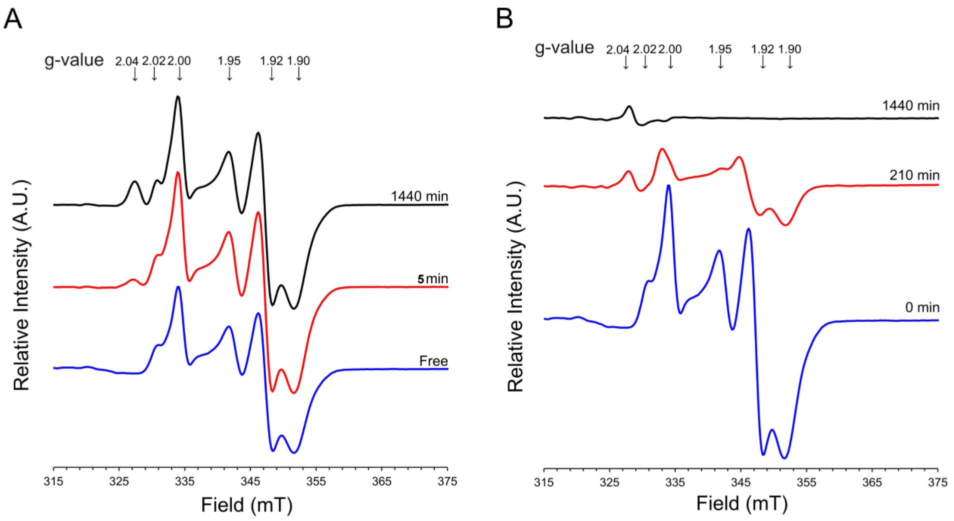

Figure S5: (**A**) EPR spectra acquired on 1:1 protein:spermine NONOate mixture: free (blue), 5 minutes (red) and 1440 minutes (black); (**B**) EPR spectra acquired on 1:2 protein:spermine NONOate mixture. Different time reaction points are shown: free (blue), 5 minutes (red) and 1440 minutes (black); At sub-stoichiometric amounts of NO, a new feature at a 327.3 mT (*g*=2.04), whose intensity increases with time, is observed. This signal has been previously reported in literature as due to thiolate-ligated DNICs in proteins, in agreement with what we observed by UV-Vis absorption spectroscopy. At higher NO concentrations, the EPR spectrum changes drastically over time *i.e.,* all features that are characteristics of reduced CISD3 disappear whereas the peaks at *g* value of 2.04 is still present.

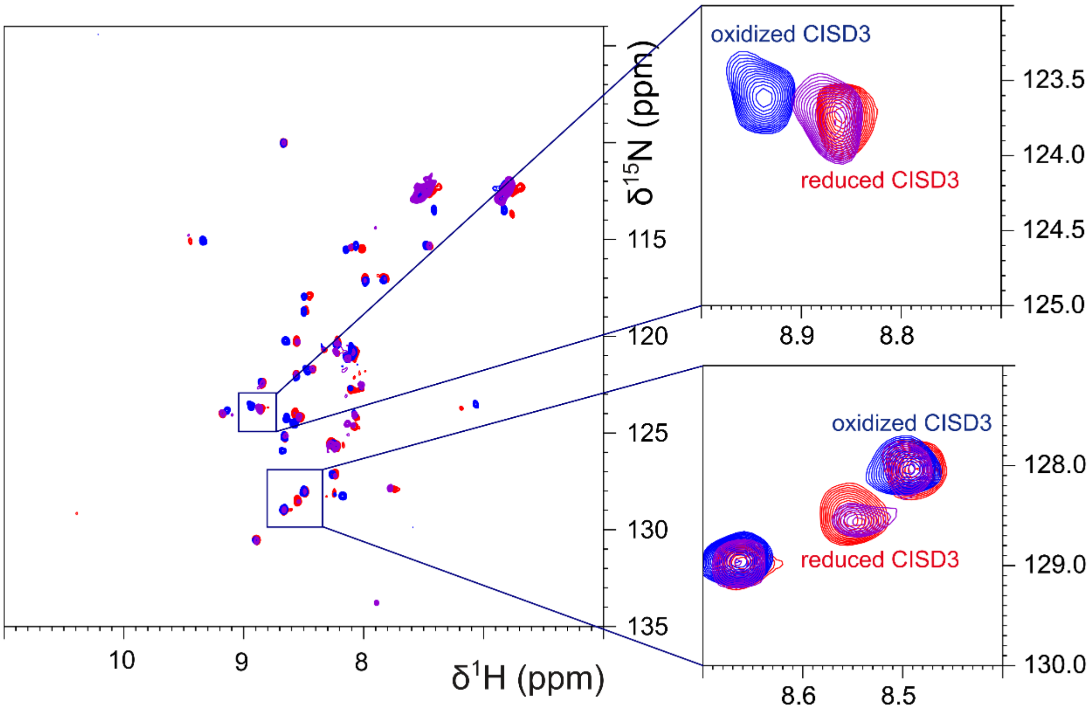

Figure S6: Interaction of oxidized CISD3 with nitric oxide studied by NMR. Overlay of the ^{1}H-^{15}N-HSQC of reduced 15N-CISD3 (red, 300 µM) with oxidized CISD3 (blue spectrum) and oxidized CISD3 with about 600 µM of nitric oxide (purple).
